# The effectiveness of a knowledge graph-based lifestyle intervention recommendation system in improving body composition, muscle strength, and physical function in sarcopenic obesity: Study protocol for a randomized controlled trial

**DOI:** 10.1371/journal.pone.0353945

**Published:** 2026-07-27

**Authors:** Xiaohong Liu, Yuxuan Lu, Meiling Fu, Zhenzhen Huang, Ting Shi, Jie Zou, Yinqin Zhong, Yun Chen

**Affiliations:** 1 Shenzhen Hospital (Futian) of Guangzhou University of Chinese Medicine, Shenzhen, China; 2 The Sixth Clinical Medical College, Guangzhou University of Chinese Medicine, Shenzhen, China; University of Padua: Universita degli Studi di Padova, ITALY

## Abstract

**Background:**

Sarcopenic obesity (SO) is a complex condition characterized by the coexistence of sarcopenia and obesity, with pathophysiological mechanisms extending beyond the additive effects of either condition alone. Lifestyle intervention is considered an effective management strategy for SO; however, substantial interindividual variability in physiological status and behavioral preferences limits the implementation of personalized intervention. This study aims to evaluate the effectiveness of a knowledge graph-based lifestyle recommendation system in improving skeletal muscle mass and related outcomes in patients with SO.

**Methods:**

This randomized controlled trial will recruit 208 patients with SO, who will be randomly assigned in a 1:1 ratio to an intervention or control group. In addition to routine lifestyle guidance, the intervention group will receive a 24-week personalized intervention delivered through the Sarcopenic Obesity-Knowledge Graph Care System (SO-KGCS), whereas the control group will receive routine health education and lifestyle advice only. The intervention period is defined as the 24-week intervention period from baseline to week 24. The primary outcome measure is skeletal muscle mass. Secondary outcome measures include grip strength, skeletal muscle mass index, body fat percentage, physical function, and quality of life. Outcomes will be assessed at baseline before randomization and at weeks 8, 16, and 24 during the 24-week intervention period. The intervention will end at week 24, after which participants will enter a 24-week post-intervention follow-up period without systematic intervention. Follow-up outcome data will be collected at week 48.

**Discussion:**

This study will evaluate the effectiveness of a knowledge graph-based recommendation system in enhancing body composition, muscle strength, physical function, and quality of life in patients with SO. If effective, the SO-KGCS may provide a feasible intelligent approach for personalized chronic disease management.

**Trial registration:**

ChiCTR, ChiCTR2500111882.

## Introduction

Sarcopenic obesity (SO) is characterized by the coexistence of sarcopenia and obesity, representing a distinct clinical condition rather than a simple combination of the two disorders. The interaction between excess adiposity and impaired muscle mass or function increases the risk of adverse health outcomes. In 2022, the European Society for Clinical Nutrition and Metabolism (ESPEN) and the European Association for the Study of Obesity (EASO) published an expert consensus defining SO as a clinical and functional disorder characterized by increased body fat mass accompanied by reduced skeletal muscle mass or function [1]. Sedentary lifestyle, dysregulated fat metabolism, and metabolic changes associated with acute or chronic comorbidities are important contributors to the development of SO [2]. Research indicates that sarcopenia affects approximately 43% of overweight or obese women and 42% of overweight or obese men [3]. In the United States, the prevalence of SO was reported to be 8.1% among individuals aged 20–59 years and 28.3% among those aged ≥60 years. The prevalence further increased in the presence of metabolic abnormalities, reaching 19.7% in prediabetes, 34.5% in type 2 diabetes, and 25.4% in metabolic-associated fatty liver disease with fibrosis [4]. In a study involving 730 children and adolescents aged 4–14 years, using the muscle mass-to-fat mass ratio based on the third BMI quintile as the diagnostic cutoff (1.22 kg/kg for females and 1.35 kg/kg for males), the prevalence of SO was 9.3% in girls and 7.2% in boys [5].

The metabolic impact of SO exceeds that of sarcopenia or obesity alone. In SO, excess adipose tissue stores large amounts of fatty acids, promoting the accumulation of proinflammatory cells within adipose tissue and creating an inflammatory milieu that induces insulin resistance, mitochondrial dysfunction, and oxidative stress [6]. In skeletal muscle, these processes trigger inflammatory and oxidative stress responses, accelerate muscle protein degradation and myocyte apoptosis, and promote muscle catabolism and impaired regeneration, ultimately leading to sarcopenia. Muscle cell injury further reduces physical performance and physical activity, worsening obesity and establishing a vicious cycle that increases the risk of metabolic diseases and functional impairment [2]. SO significantly raises the risk of heart disease, metabolic syndrome, dementia, and postoperative complications. It also substantially elevates the risk of frailty, disability, and depression, with 2.7-fold and 2.9-fold higher risks of falls and disability, respectively [7–9], and a 24% increase in all-cause mortality [10]. Consequently, SO imposes a considerable burden on public health, families, and society. Once established, SO may persist throughout life, underscoring the importance of early recognition and intervention to reduce adverse metabolic outcomes. However, most patients with SO are diagnosed only after severe functional impairment caused by muscle loss has already occurred [11], resulting in missed opportunities for optimal early treatment.

Management of SO focuses on improving muscle mass while reducing excess adiposity. However, sarcopenia and obesity are currently treated as separate clinical conditions [10]. In elderly patients with SO, this separation creates challenges in dietary management because the need for increased protein intake may conflict with calorie restriction goals [12]. Similar challenges arise when combining resistance training to increase muscle mass with aerobic exercise aimed at fat reduction. In addition, substantial interindividual differences in physical condition, disease status, and metabolic rate make it difficult to design individualized dietary, nutritional, and exercise interventions. Effective management, therefore, requires collaboration among multiple professional teams, making clinical implementation complex and time-consuming.

Internet-based recommendation systems offer a novel strategy for personalized health interventions [13]. Health recommendation systems are specialized recommendation systems that provide users with personalized health information by automatically identifying and recommending appropriate content based on individual health conditions and needs [14]. In recent years, knowledge graph-based recommendation systems have been widely applied in domains such as movies, music, news, and product recommendations [15–17]. By incorporating knowledge graphs as auxiliary information, these systems effectively address the sparsity and cold-start limitations of traditional recommendation systems. Knowledge graph-based models can mine relational data and integrate it with existing user and item information, increasing data dimensionality and improving recommendation accuracy. Although recommendation systems have long been studied in healthcare, their application in SO management remains limited.

Based on this, we designed a randomized controlled trial to evaluate the effectiveness of a knowledge-graph-based care recommendation system in improving body composition, muscle strength, and physical function among patients with SO. We hypothesize that patients receiving this intelligent system-based intervention will achieve greater clinical benefits than those receiving standard care, thereby providing new evidence-based support and a feasible strategy for precision and intelligent management of chronic diseases in the elderly.

## Methods

### Study design

**Fig 1:**
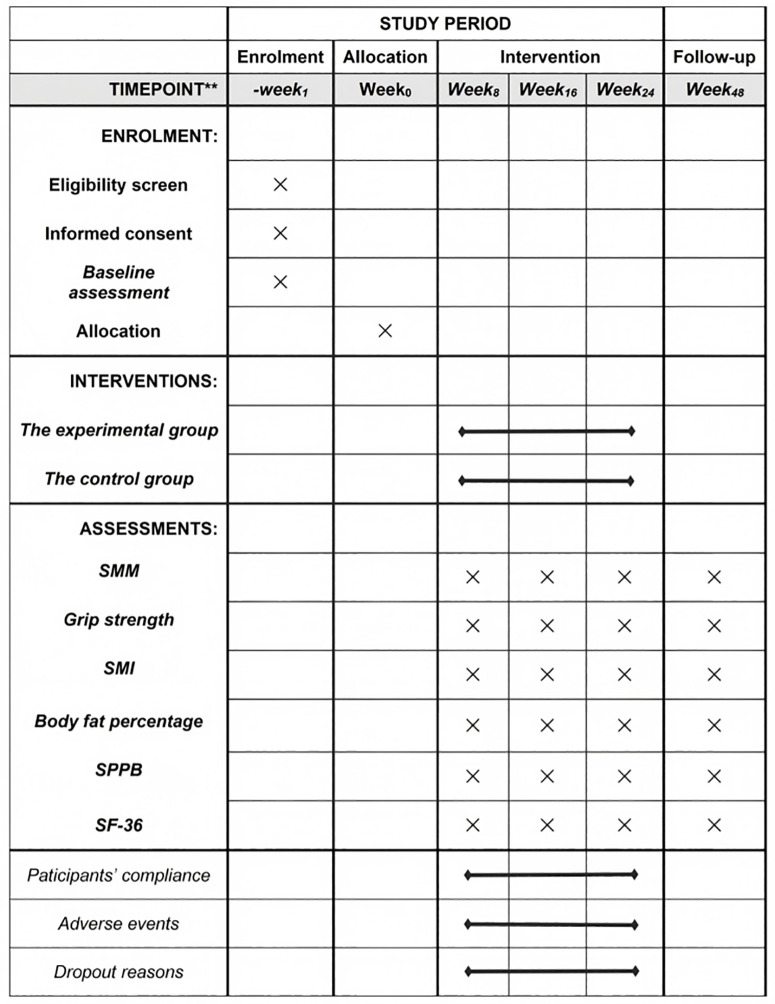
Enrollment, interventions, and assessments schedule SMM, skeletal muscle mass; SMI, skeletal muscle index; SF-36, 36-item short form health survey.

**Fig 2:**
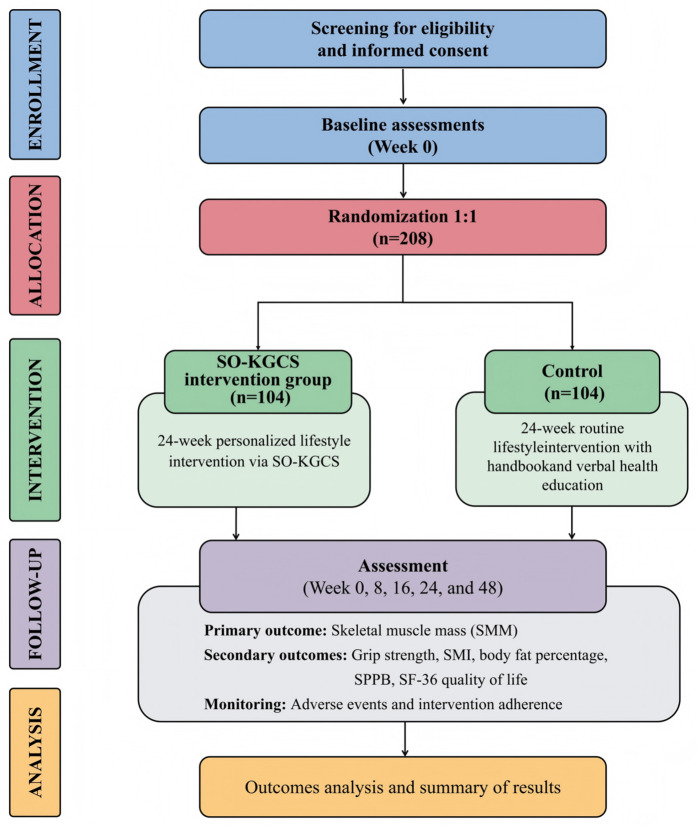
Study flow chart.

This single-blind, two-arm randomized controlled trial is designed to evaluate the effects of a knowledge graph-based lifestyle recommendation system on skeletal muscle mass, grip strength, skeletal muscle mass index, body fat percentage, physical function, and quality of life in patients with SO. Participants will be randomly assigned in a 1:1 ratio to either the intervention group or the control group. The intervention group will receive a 24-week lifestyle intervention aimed at increasing muscle mass and reducing weight, while the control group will receive conventional treatment and health education without personalized recommendations from the system. The total study duration is 48 weeks, comprising a 24-week intervention period followed by a 24-week follow-up period. Assessments will be conducted at baseline and at weeks 8, 16, 24, and 48, with week 24 marking the end of the intervention. Thereafter, both groups will enter a follow-up phase without systematic intervention and will be observed until week 48. The randomization sequence will be generated by a statistician using a computer-generated block randomization with block sizes of 4 or 6. Allocation concealment will be ensured through a password-protected centralized network system accessible only to independent registrars not involved in outcome assessment after completion of baseline assessments. Because of the nature of the lifestyle intervention, blinding of participants and intervention implementers is not possible. However, outcome assessors and statistical analysts will remain blinded throughout the study. All outcome assessments will be completed by uniformly trained independent assessors unaware of group allocation. Data managers and statistical analysts will remain blinded until database lock. Participants will be instructed not to disclose their group allocation to assessors, and an emergency unblinding procedure will be established if required. Intervention reporting will follow the Template for Intervention Description and Replication (TIDieR) checklist. The study protocol is developed in accordance with the SPIRIT statement (S1 File). Fig 1 and Fig 2 illustrate the proposed study timeline and procedures.

### Participants

Healthcare professionals will recruit participants through the Department of Endocrinology at Shenzhen Hospital (Futian) of Guangzhou University of Chinese Medicine. Recruitment announcements will be posted on local social media platforms (WeChat, TikTok, Xiaohongshu). In addition, primary care centers will distribute informational pamphlets, and meetings will be held with prospective participants.

### Inclusion criteria

Participants will be eligible if they meet all of the following criteria:

1) SO was diagnosed based on the concurrent presence of sarcopenia, obesity, and adequate baseline functional capacity [18]. Sarcopenia will be defined according to the Asian Working Group for Sarcopenia (AWGS) 2019 criteria, requiring low muscle strength alongside either reduced physical performance or low muscle mass. Low muscle strength will be defined as handgrip strength <28 kg for men and <18 kg for women. Reduced physical performance or low muscle mass will be defined as a Short Physical Performance Battery (SPPB) score ≤9, or an appendicular skeletal muscle mass index (ASMI) <7.0 kg/m^2^ in men and <5.4 kg/m^2^ in women, assessed using dual-energy X-ray absorptiometry (DXA) [19]. Obesity will be defined as either a body mass index (BMI) ≥28 kg/m^2^ or a body fat percentage >25% in men and >30% in women, measured using the InBody 770 device [20].
2) Age ≥ 60 years;
3) Ability to safely participate in the intervention;
4) Willingness to undergo study assessments and provision of written informed consent voluntarily.

### Exclusion criteria

Participants meeting any of the following criteria will be excluded:

1) Acute myocardial infarction, unstable angina, stroke, heart failure, or other severe cardiovascular or cerebrovascular diseases that preclude participation in exercise training or physical function assessments;
2) Severe hepatic or renal insufficiency, uncontrolled severe endocrine disorders, active malignancy, or current receipt of radiotherapy;
3) Diabetes mellitus;
4) Severe psychiatric disorders, cognitive impairment, or any other condition that would impair compliance with the intervention or follow-up;
5) Severe osteoarticular disease causing marked limitation of motor function;
6) Current participation in another clinical trial.

### Discontinuation of the intervention and withdrawal from the study

Participants may discontinue the intervention or withdraw from the study at any time. Discontinuation of the intervention will not be regarded as withdrawal from the study unless the participant explicitly requests withdrawal or declines further follow-up. Participants who discontinue the intervention will be encouraged to complete all scheduled follow-up assessments whenever possible. The intervention may be temporarily suspended or permanently discontinued at the investigator’s discretion under any of the following circumstances:

1) occurrence of a serious adverse event, an adverse event considered related to the intervention, or any other condition that makes continued intervention unsafe;
2) significant deterioration in key health indicators or the development of clinical conditions that make continued intervention inappropriate;
3) persistent inadequate adherence despite reminders and assessment of possible causes, defined as weekly adherence of <50% for two consecutive weeks or overall adherence of <50% during the study period;
4) inability to understand or follow the intervention recommendations or a newly identified or previously undisclosed medical condition that may compromise intervention safety;
5) a request by the participant to discontinue the intervention or withdraw from the study.

For participants who discontinue the intervention or withdraw from the study, investigators will make reasonable efforts, with the participant’s consent, to document the reason for and date of discontinuation or withdrawal and to complete feasible safety and outcome assessments. Appropriate clinical management will be provided if discontinuation is related to adverse events, deterioration in health status, or unsatisfactory treatment effects.

All available data collected before discontinuation or withdrawal will be included in the analysis unless the participant withdraws consent for data use. No replacement participants will be recruited. All randomized participants will be analyzed according to the intention-to-treat (ITT) principle, regardless of intervention discontinuation, protocol deviations, or adherence level. Protocol deviations, unresolved data-quality issues, and adherence-related issues will be documented and considered in sensitivity or per-protocol analyses, as appropriate.

### Sample size

The sample size was calculated using G-power 3.1 software. The calculation was based on a two-sided comparison of the mean change in skeletal muscle mass (SMM) between the two groups, with SMM prespecified as the sole primary confirmatory outcome. The expected effect size was derived from a previous Internet-based nutrition and exercise intervention study in older adults with sarcopenia [21]. In that study, the mean change in SMM was −0.10 kg in the control group and 1.09 kg in the comprehensive intervention group, corresponding to a between-group difference of 1.19 kg. Because the standard deviation of the SMM change score was not directly reported, it was conservatively estimated from the reported baseline and post-intervention standard deviations, assuming a pre-post correlation coefficient of 0.50. This yielded an estimated pooled standard deviation of approximately 2.70 kg and a Cohen’s d of approximately 0.44. Although the proposed intervention incorporates a knowledge graph-based recommendation system that may enhance intervention individualization, this moderate effect size was adopted to avoid overestimating the intervention effect. With a two-sided α level of 0.05, power of 0.80, and a 1:1 allocation ratio, 83 participants were required per group. Factoring an anticipated dropout rate of 20%, 104 participants will be enrolled in each group, resulting in a total sample size of 208 participants.

### Procedure

Potential participants will first undergo screening to determine eligibility, including assessment of medical history and lifestyle habits. Individuals meeting the eligibility criteria will be invited to an in-person visit, during which the study procedures will be explained and written informed consent will be obtained.

All baseline assessments will be completed on a single day following participant enrolment. Upon arrival at the hospital, participants will complete questionnaires collecting demographic and clinical information, including sex, age, height, weight, marital status, home address, and contact information. Body composition measurements and physical fitness tests will then be performed to assess mobility, muscle strength, and overall physical function. Participants will also complete supplementary questionnaires at home covering dietary status, quality of life, and SO-related symptoms.

After baseline assessment, trained research staff will assist participants in the intervention group with downloading and using the SO-KGCS system. Based on baseline assessment results, the system will provide personalized plans for nutrition, exercise, and lifestyle modification. Participants in the intervention group will receive regular personalized updates through the system, while those in the control group will receive standard health education. The intervention period will last 24 weeks. During the study, all participants will be instructed to maintain a daily calorie intake approximately 200 kcal below their baseline levels, in accordance with the ESPEN and EASO Consensus Statement on SO. Daily protein intake will be maintained at ≥1.2 g/kg body weight.

All assessments will be performed by uniformly trained professional nurses using standardized procedures. After baseline assessment and randomization at week 0, outcome assessments will be conducted at weeks 8, 16, and 24, with week 24 defined as the end of the intervention. A final follow-up assessment will be conducted at week 48, corresponding to 24 weeks after completion of the intervention. Adherence, adverse events, and reasons for withdrawal will be recorded throughout the intervention period.

### Randomization and blinding

Participants will be randomized in a 1:1 ratio to the intervention or control group using a computer-generated block randomization sequence prepared by an independent statistician, using random blocks of 4 or 6 to prevent prediction of assignment. Allocation concealment will be maintained through a password-protected centralized web-based system accessible only to an independent registrar after completion of baseline assessments. Because of the nature of the personalized lifestyle intervention, participants and intervention providers cannot be blinded. However, outcome assessors and statisticians will remain blinded throughout the study. Outcome assessments will be completed by standardized independent assessors unaware of group allocation. Participants will be instructed not to disclose their group allocation to assessors. Intervention providers will not participate in outcome assessments. An emergency unblinding protocol supervised by the Ethics Committee will also be established to address potential safety risks.

### Intervention

#### Control group intervention.

Participants in the control group will receive a routine lifestyle intervention. Following hospital discharge or outpatient visits, each participant will receive an SO lifestyle management handbook and verbal guidance on basic SO management in older adults, including the risks associated with sarcopenia and obesity, dietary recommendations such as increasing protein intake and reducing high-fat and high-sugar foods, exercise advice including daily 30-minute walks and simple limb activities, and chronic disease management measures such as ensuring adequate sleep and monitoring blood pressure and blood glucose levels.

### Experimental group

#### Preliminary preparation.

**Fig 3:**
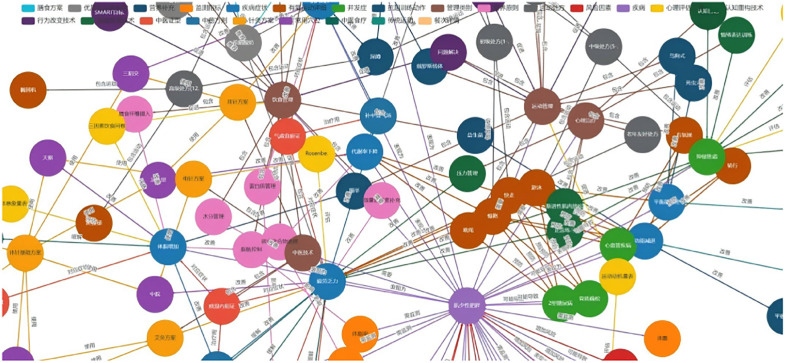
Knowledge Map of SO.

A systematic search was conducted across multiple databases and relevant websites, including BMJ Best Practice, UpToDate, the Joanna Briggs Institute (JBI, Australia), the Center for Evidence-Based Healthcare, the Guidelines International Network (GIN), the Cochrane Library, OVID, PubMed, Web of Science, the European Society for Clinical Nutrition and Metabolism, the Academy of Nutrition and Dietetics, the Society for Parenteral and Enteral Nutrition, MedLink, the China Biomedical Literature Service Network, CNKI, Wanfang, and VIP, to retrieve lifestyle-related content concerning SO. The search period extended from database inception to August 1, 2025. Based on the retrieved literature, the research team developed a preliminary lifestyle intervention plan for SO. Following expert review and revision by a multidisciplinary panel of 10 endocrinologists, nurses, and nutritionists, and considering Chinese national conditions and patient needs, the final intervention plan and knowledge graph were established (Fig 3).

The knowledge map integrates multidimensional information such as disease characteristics, nutritional requirements, exercise modalities, traditional Chinese medicine (TCM) interventions, and lifestyle factors, thereby forming a structured intervention knowledge base to support personalized recommendations.

#### Intervention implementation.

**Fig 4:**
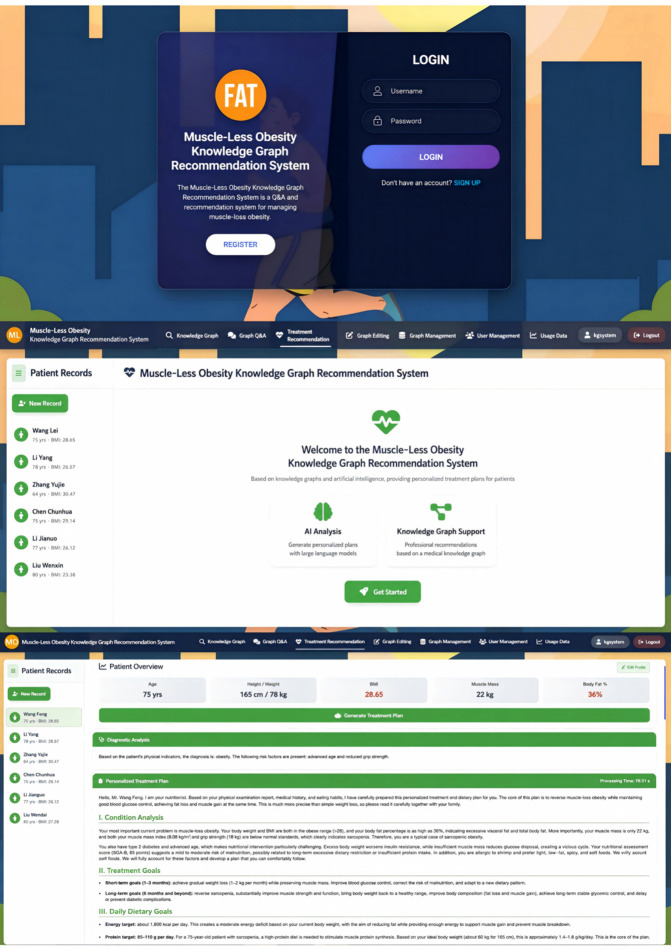
Knowledge Graph-Based Recommendation System.

In addition to the routine lifestyle guidance for SO, participants in the intervention group will be instructed to use the Sarcopenic Obesity-Knowledge Graph Care System (SO-KGCS) for personalized lifestyle intervention. SO-KGCS is a mobile application developed specifically for patients with SO. Built on a knowledge graph framework, the system integrates multisource data to identify patterns of coexisting low muscle mass and excess fat accumulation, and generates personalized nutrition, exercise, and lifestyle recommendations aimed at simultaneous fat reduction and muscle gain. After logging in, participants will enter the treatment advice interface and complete personal information, including demographic characteristics, functional indicators, physiological and biochemical parameters, medical history, medication history, and lifestyle habits. Based on these data, the system will automatically generate a personalized intervention plan comprising disease assessment, treatment objectives, exercise training, dietary modification, TCM interventions, lifestyle optimization, and necessary medical recommendations, together with expected outcomes and precautions. The feedback section will allow patients to report their experiences, enabling medical staff to adjust recommendations when necessary. Participants will be required to follow the recommended lifestyle modifications and regularly report their progress through the platform. The system interface is shown in Fig 4.

To ensure intervention fidelity and minimize confounding, permitted and prohibited concomitant treatments during the study period have been defined. Participants may continue stable existing medications and receive necessary emergency care. However, participation in other structured programs targeting weight loss, muscle gain, or physical function improvement, as well as unauthorized use of medications affecting muscle metabolism or body composition, will be prohibited. Compliance will be monitored through regular follow-up, and all relevant events will be documented and considered in the analysis.

### Strategies to improve intervention adherence

To enhance intervention adherence, the research team will provide close monitoring, regular targeted clinical guidance, biweekly follow-up visits, and supportive education through mobile applications. Family members or other individuals within the participant’s social network will also be involved at study initiation to support recommended lifestyle modifications. To improve data completeness and maintain long-term follow-up validity, several strategies will be implemented, including obtaining informed consent for long-term follow-up at enrollment, reducing participant burden through optimized procedures such as telephone follow-up and online questionnaires, maintaining engagement through regular health newsletters and appropriate incentives, and establishing a multi-channel contact mechanism with backup contact information to track participants lost to follow-up. Appropriate statistical methods will be used to manage and report missing follow-up data.

### Adverse events

All adverse events occurring during the study will be recorded and monitored. Data will be collected on falls, injuries, musculoskeletal problems, hypoglycemic episodes, major cardiovascular disease events, and any other events potentially related to the study procedures or interventions. All adverse events will be carefully evaluated and followed until symptoms or signs resolve or stabilize. Detailed records of management measures and outcomes will be maintained. Follow-up methods will be selected according to event severity and may include inpatient observation, outpatient follow-up, home visits, telephone follow-up, or written communication.

### Measures

#### Primary outcome measures.

The primary outcome measure of this study is skeletal muscle mass (SMM, kg) assessed using bioelectrical impedance analysis (BIA). Before measurement, participants will be instructed to avoid strenuous exercise, alcohol consumption, and excessive fluid intake, and to empty their bladders. Measurements will be performed under resting conditions according to a standardized protocol. Participants will stand barefoot on the electrode plates of the body composition analyzer, hold the hand electrodes with both hands, maintain an upright posture, and keep the upper limbs naturally abducted without touching the trunk. All measurements will be conducted by trained researchers during the same time period and under consistent environmental conditions to minimize the influence of diet, exercise, and hydration status. SMM will be recorded in kilograms (kg) to one decimal place.

#### Secondary outcome measures.

1) Grip Strength

Grip strength will be measured using a dynamometer (Jamar Plus+, USA) and recorded in kilograms. Participants will adjust the grip width to a comfortable position and perform maximal voluntary contraction for 3 seconds. Three measurements will be obtained for each hand and the highest value will be recorded. If the variation between measurements exceeds 10%, participants will rest for 10–15 minutes before repeat testing.

2) Skeletal Muscle Mass Index

Skeletal muscle mass index (SMI, kg/m²) will be assessed as a secondary outcome. Appendicular skeletal muscle mass (ASM, kg) will be measured using BIA with the same analyzer used for SMM assessment. Measurement procedures and precautions will be identical to those described for the primary outcome. SMI will be calculated as ASM divided by height squared: SMI = ASM (kg)/ height² (m²). Results will be expressed in kg/m² and recorded to two decimal places.

3) Body Fat Percentage

Body fat percentage will be measured using the same body composition analyzer under standardized conditions as described above. Results will be expressed as percentages (%) and recorded to one decimal place.

4) Physical Function Assessment

Physical function will be assessed using the Short Physical Performance Battery (SPPB), which includes a balance test, a 4-meter usual gait speed test, and a five-times sit-to-stand test. These components assess balance, walking ability, lower-limb muscle strength, and endurance, respectively. The balance test includes side-by-side, semi-tandem, and tandem standing positions. Each component will be scored from 0 to 4, yielding a total score ranging from 0 to 12, with higher scores indicating better physical function.

5) Quality of Life

Quality of life will be assessed using the MOS 36-item Short Form Health Survey (SF-36) [22]. The SF-36 evaluates eight domains of health-related quality of life: Physical Functioning (PF), Role-Physical (RP), Bodily Pain (BP), General Health (GH), Vitality (VT), Social Functioning (SF), Role-Emotional (RE), and Mental Health (MH). Scores for each domain will be converted to a 0–100 scale using the following formula: Converted Score = (Actual Score − Minimum Score for that Dimension)/ (Maximum Score for that Dimension − Minimum Score for that Dimension) × 100. The mean score for the eight domains will be calculated as the composite score, with higher scores indicating better quality of life.

### Statistical analysis

The primary efficacy analysis will be conducted according to the ITT principle, with all randomized participants analyzed in the groups to which they were originally assigned. Continuous variables will be summarized as means ± standard deviations or medians with interquartile ranges, as appropriate based on distribution, and categorical variables will be presented as frequencies and percentages. Baseline characteristics will be summarized by treatment group. Between-group comparisons at baseline will be performed using independent-samples *t* tests or Mann-Whitney U tests for continuous variables, and χ² tests or Fisher’s exact tests for categorical variables.

The primary analysis population will be the ITT population. A per-protocol set analysis will be conducted as a sensitivity analysis and will include participants who complete the intervention and follow-up assessments, meet prespecified adherence criteria, and have no major protocol deviations. Poor adherence will be defined as an intervention adherence rate of <50% per week for two consecutive weeks or an overall intervention adherence rate of <50% during the study period. The safety analysis population will include participants who receive at least one intervention session or complete at least one post-randomization safety assessment.

The sole primary confirmatory outcome will be SMM. The primary intervention effect will be defined as the adjusted between-group difference in change in SMM from baseline to the end of the 24-week intervention. The primary outcome will be analyzed using a linear mixed-effects model, with post-randomization SMM measurements as the dependent variable. Fixed effects will include group, time, the group-by-time interaction, and prespecified covariates, including age, sex, body weight, and baseline SMM. A participant-level random intercept will account for within-participant correlation arising from repeated measurements. Time will be modeled as a categorical variable to avoid imposing a linear change trajectory. The primary effect will be estimated from the group-by-time interaction at week 24, and the adjusted between-group mean difference, 95% confidence interval, and two-sided P value will be reported.

Secondary outcomes will include handgrip strength, skeletal muscle index, body fat percentage, SPPB score, and SF-36 quality-of-life score. Each secondary outcome will be analyzed using a linear mixed-effects model analogous to that used for the primary outcome. Fixed effects will include group, time, the group-by-time interaction, and prespecified covariates, including age, sex, body weight, and the baseline value of the corresponding outcome. Adjusted between-group differences, 95% confidence intervals, and two-sided P values will be reported. SMM will be the only primary confirmatory outcome, whereas secondary outcomes will provide supportive evidence regarding intervention effects. For analyses involving multiple secondary outcomes and repeated time points, the Benjamini–-Hochberg procedure will be applied to control the false discovery rate.

All available repeated-measures data will be included in the linear mixed-effects models using maximum likelihood estimation under the missing-at-random assumption. The extent, timing, and pattern of missing data, together with reasons for withdrawal or intervention discontinuation, will be summarized by group. For the primary outcome, a sensitivity analysis will be conducted using multiple imputation by chained equations. The imputation model will include randomized group, age, sex, height, body weight, BMI, baseline SMM, available follow-up SMM measurements, and other variables potentially associated with missingness or the primary outcome. Twenty imputed datasets will be generated and analyzed using the primary analysis model, with estimates pooled according to Rubin’s rules. If missingness in the primary outcome is substantial or the missing-data mechanism remains uncertain, a tipping-point analysis based on delta-adjusted multiple imputation will be performed. In this analysis, a series of prespecified unfavorable shifts will be applied to the imputed SMM values to assess the robustness of the primary conclusions under plausible missing-not-at-random assumptions.

Prespecified exploratory subgroup analyses will be performed by age and sex through inclusion of group-by-time-by-subgroup interaction terms in the linear mixed-effects model. Model assumptions will be evaluated using residual plots, normal Q-Q plots, and outlier diagnostics. If substantial heteroscedasticity or deviation from residual non-normality is detected, robust standard errors will be used in sensitivity analyses while retaining the primary model structure. Safety analyses will be primarily descriptive. Adverse events and serious adverse events will be summarized by group, severity, possible relationship to the intervention, and outcome. All statistical tests will be two-sided, and *P* < 0.05 for the primary outcome will be considered statistically significant. Statistical analyses will be performed using R software, version 4.4.2 (R Foundation for Statistical Computing, Vienna, Austria).

### Data management and quality control

Strict data management and quality control measures will be implemented to ensure data reliability and participant confidentiality. All study data will be collected using case report forms (CRFs). Two uniformly trained researchers will independently enter the data into an encrypted electronic database. Data will then be checked for completeness, accuracy, and logical consistency, and any discrepancies will be verified against the original CRFs. All physical assessments and body composition measurements will be performed by the same trained physician using the same equipment. Data quality checks will be conducted immediately following each assessment to minimize measurement errors. All paper and electronic study documents will be securely stored. The study will strictly adhere to the principles of confidentiality, and all personally identifiable information will be replaced with research codes to ensure full protection of participant privacy.

### Ethics and dissemination

The original study protocol (version 1.0, dated July 29, 2025) was approved by the Research Ethics Committee of Shenzhen Hospital (Futian), Guangzhou University of Chinese Medicine on September 10, 2025 (Approval No. GZYLL(KY)-2025−107). Before trial initiation and statistical analysis, several protocol clarifications and methodological refinements were introduced to improve the consistency, transparency, and interpretability of the study. These refinements primarily involved standardization of study time points, outcome assessment, and the statistical analysis plan. The revised protocol (version 2.0, dated April 28, 2026) was approved by the same ethics committee on May 14, 2026 (Revision Approval No. GZYLL(KY)-2025-107-01). The updated protocol and relevant approval documents are provided as supporting information. This study will be conducted in accordance with the principles of the Declaration of Helsinki. Written informed consent will be obtained from all participants before enrollment. Any future modifications to assessment methods, eligibility criteria, or study management procedures will require ethics committee approval before implementation. Upon completion of the study, the findings will be submitted for publication in a peer-reviewed journal.

### Trial status

This trial has been registered with the Chinese Clinical Trial Registry (ChiCTR2500111882). Participant recruitment began on January 1, 2026, and is expected to be completed by September 1, 2026. The trial is currently recruiting participants. The anticipated study completion date is September 2027.

## Discussion

This work presents a randomized controlled trial protocol evaluating the effectiveness of a knowledge graph-based care recommendation system (SO-KGCS) for personalized management of SO. The health burden of SO extends beyond the additive effects of sarcopenia and obesity alone. However, current clinical management remains limited by conflicts between dietary and exercise interventions [12,23], and the lack of precise individualized treatment strategies, resulting in missed opportunities for early intervention. Knowledge graph technology has been widely applied in personalized recommendation systems across multiple fields. In online education, integrating knowledge graphs with collaborative filtering and semantic embedding techniques has significantly improved course recommendation accuracy and recall [23]. In healthcare, combining knowledge graphs with deep learning has enhanced disease prediction and intervention recommendation performance, demonstrating considerable potential for intelligent healthcare [24]. Building on these advances, the present study will incorporate knowledge graph technology by integrating medical evidence and clinical data to identify co-occurrence patterns of low muscle mass and fat accumulation and dynamically generate personalized intervention plans, thereby addressing limitations of conventional manual management. The core innovation of this study is the development and application of the SO-KGCS system, which integrates multidisciplinary recommendations involving nutrition, exercise, TCM interventions, and lifestyle modifications. The system supports real-time feedback and dynamic adjustment of intervention plans, facilitating a shift from standardized care to personalized precision management. In addition, family involvement and regular follow-up strategies are incorporated to improve adherence. The study also emphasizes feasibility and safety through clearly defined eligibility criteria, continuous adverse event monitoring, and the use of clinically relevant and operational outcome measures to comprehensively evaluate intervention effectiveness.

## Limitations

This study has several limitations. First, participants will be recruited primarily from a single medical center, which may limit sample representativeness and reduce the generalizability of the findings. Second, the relatively short intervention and follow-up periods may limit evaluation of the long-term impact of the knowledge graph-based recommendation system on SO-related metrics. Third, differences in lifestyle guidance between the intervention and control groups may introduce performance bias despite the randomized controlled design. Finally, subjective assessments and measurement variability in indicators such as body composition and muscle strength may affect result accuracy. Future studies should address these limitations through multicenter recruitment, longer intervention durations, and extend follow-up periods.

## Conclusion

This study has important academic and clinical implications. To our knowledge, this will be the first randomized controlled trial to evaluate a knowledge graph-based care recommendation system for SO intervention. The findings are expected to provide evidence-based support for the application of intelligent technologies in chronic disease management and expand the evidence base for SO interventions. If proven effective, the SO-KGCS could be integrated into clinical practice to improve the efficiency and accessibility of precision interventions, reduce the workload of multidisciplinary teams, support early reversal of pathological changes, and lower the risks of adverse outcomes such as cardiovascular disease and frailty. The study may also offer valuable insights for the intelligent management of other chronic conditions.

## Supporting information

S1 File SPIRIT checklist. (DOCX)

S2 File Research Protocol 2.0 (Chinese and English). (DOCX)
