# Supplementary material for: The effectiveness of a knowledge graph-based lifestyle intervention recommendation system in improving body composition, muscle strength, and physical function in sarcopenic obesity: Study protocol for a randomized controlled trial
Source: PLoS One. 2026 Jul 27;21(7):e0353945. doi: 10.1371/journal.pone.0353945 (PMC13405061; doi:10.1371/journal.pone.0353945)
Supplement: S2 File — (DOCX) [file pone.0353945.s002.docx]

**Research Protocol**

**Research Project Name:** The effectiveness of a knowledge graph-based lifestyle intervention recommendation system in improving body composition, muscle strength, and physical function in sarcopenic obesity: Study protocol for a randomized controlled trial

**Project Source:** Guangzhou University of Chinese Medicine “Double First-Class” Discipline High-Quality Development “Peak-Building and Excellence-Forging” Action Plan (2024-2027) (GZY2025GB0925); Shenzhen Futian District Health System Scientific Research Project (FTWS2025038); Chronic Disease Management Research Project of National Health Commission Capacity Building and Continuing Education Center (GWJJMB202510024225).

**Project Leader:** Yun Chen

**Version: V2.0**

**Version Date:** April 28th, 2026

**Researcher Statement**

As the principal investigator of this research project, I will adhere to the ethical review measures for biomedical research involving humans issued by the National Health and Family Planning Commission (2016), the World Medical Association's Declaration of Helsinki (2013), the CIOMS International Ethical Guidelines for Biomedical Research Involving Human Subjects, and the ethical principles of GCP. I will conduct the research in accordance with study protocols approved by the Medical Ethics Committee, under the guidance of the quality management standards for clinical drug trials, to ensure the scientific validity of the study and safeguard the health and rights of the participants.

Name: Yun Chen

Date: April 28th, 2026

**I.Research Background**

Sarcopenic obesity (SO) is characterized by the coexistence of sarcopenia and obesity and is recognized as a distinct clinical condition rather than a simple combination of the two disorders. In SO, excessive adiposity interacts with reduced skeletal muscle mass, impaired muscle strength, and reduced physical performance, increasing the risk of metabolic abnormalities, frailty, disability, falls, cardiovascular disease, and reduced quality of life. SO is therefore associated with a substantial burden on patients, families, healthcare services, and society.

Lifestyle intervention remains a key strategy for SO management. Effective intervention requires simultaneous improvement of muscle mass and strength and reduction of excess adiposity. However, patients with SO differ substantially in age, physical function, comorbidities, metabolic status, dietary habits, exercise capacity, and behavioral preferences. These differences make standardized or manually developed lifestyle advice difficult to individualize, implement, and sustain in routine clinical practice.

A knowledge graph-based recommendation system can integrate structured medical knowledge and multidimensional patient data, allowing individualized recommendations for nutrition, exercise, lifestyle modification, and non-invasive traditional Chinese medicine (TCM)-informed health management. This study will evaluate the effectiveness and safety of the Sarcopenic Obesity-Knowledge Graph Care System (SO-KGCS) in improving skeletal muscle mass and related outcomes in older adults with SO.

**II.Objectives and Significance of the Research**

**2.1 Primary Objective**

To evaluate whether a 24-week personalized lifestyle intervention delivered through SO-KGCS, in addition to routine lifestyle guidance, improves skeletal muscle mass (SMM) in patients with SO compared with routine lifestyle guidance alone.

**2.2 Secondary Objectives**

To evaluate the effects of SO-KGCS on grip strength, skeletal muscle mass index (SMI), body fat percentage, physical function, and health-related quality of life.

To evaluate the safety of the SO-KGCS intervention by monitoring falls, injuries, musculoskeletal symptoms, hypoglycemic episodes, major cardiovascular events, and other adverse events.

To assess intervention adherence, system usability, and the feasibility of integrating the knowledge graph-based recommendation system into SO management.

To explore whether intervention responses differ by prespecified participant characteristics such as age and sex. These analyses will be exploratory and will not determine the primary efficacy conclusion.

**2.3 Significance**

This study will provide clinical evidence on whether an intelligent lifestyle recommendation system can support precise, feasible, and safe chronic disease management for SO. If effective, SO-KGCS may reduce the workload of multidisciplinary teams and improve the accessibility of individualized lifestyle interventions for older adults with SO.

**III. Research Methods**

**3.1 Study Design**

This single-blind, two-arm randomized controlled trial is designed to evaluate the effects of a knowledge graph-based lifestyle recommendation system on skeletal muscle mass, grip strength, skeletal muscle mass index, body fat percentage, physical function, and quality of life in patients with SO. Participants will be randomly assigned in a 1:1 ratio to either the intervention group or the control group. The intervention group will receive a 24-week lifestyle intervention aimed at increasing muscle mass and reducing weight, while the control group will receive conventional treatment and health education without personalized recommendations from the system. The total study duration is 48 weeks, comprising a 24-week intervention period followed by a 24-week follow-up period. Assessments will be conducted at baseline and at weeks 8, 16, 24, and 48, with week 24 marking the end of the intervention. Thereafter, both groups will enter a follow-up phase without systematic intervention and will be observed until week 48. The randomization sequence will be generated by a statistician using a computer-generated block randomization with block sizes of 4 or 6. Allocation concealment will be ensured through a password-protected centralized network system accessible only to independent registrars not involved in outcome assessment after completion of baseline assessments. Because of the nature of the lifestyle intervention, blinding of participants and intervention implementers is not possible. However, outcome assessors and statistical analysts will remain blinded throughout the study. All outcome assessments will be completed by uniformly trained independent assessors unaware of group allocation. Data managers and statistical analysts will remain blinded until database lock. Participants will be instructed not to disclose their group allocation to assessors, and an emergency unblinding procedure will be established if required.

Intervention reporting will follow the Template for Intervention Description and Replication (TIDieR) checklist, and the protocol is developed in accordance with the SPIRIT statement. The proposed study timeline and procedures are presented in the enrollment, intervention, assessment schedule and study flow chart.

**3.2 Research venue**

Participants will be recruited and assessed through the Department of Endocrinology at Shenzhen Hospital (Futian), Guangzhou University of Chinese Medicine, with support from collaborating primary care centers when appropriate.

**3.3 Study Subjects**

**3.3.1 Calculation of Sample Volumes**

The sample size was calculated using G-power 3.1 software. The calculation was based on a two-sided comparison of the mean change in skeletal muscle mass (SMM) between the two groups, with SMM prespecified as the sole primary confirmatory outcome. The expected effect size was derived from a previous Internet-based nutrition and exercise intervention study in older adults with sarcopenia. In that study, the mean change in SMM was −0.10 kg in the control group and 1.09 kg in the comprehensive intervention group, corresponding to a between-group difference of 1.19 kg. Because the standard deviation of the SMM change score was not directly reported, it was conservatively estimated from the reported baseline and post-intervention standard deviations, assuming a pre-post correlation coefficient of 0.50. This yielded an estimated pooled standard deviation of approximately 2.70 kg and a Cohen’s d of approximately 0.44. Although the proposed intervention incorporates a knowledge graph-based recommendation system that may enhance intervention individualization, this moderate effect size was adopted to avoid overestimating the intervention effect. With a two-sided α level of 0.05, power of 0.80, and a 1:1 allocation ratio, 83 participants were required per group. Factoring an anticipated dropout rate of 20%, 104 participants will be enrolled in each group, resulting in a total sample size of 208 participants.

**3.3.2 Recruiting Strategy**

Inpatient and outpatient recruitment: Recruitment posters will be displayed in the outpatient and inpatient areas of the study center. Endocrinologists and trained healthcare professionals will identify and refer potentially eligible participants during routine clinical care.

Community recruitment: Through collaboration with community health service centers, the study team will distribute informational pamphlets and hold meetings or health education sessions for prospective participants.

Online recruitment: Recruitment announcements may be posted on institutional-authorized social media platforms, including WeChat, TikTok, and Xiaohongshu, in accordance with hospital and ethics committee requirements.

All recruitment materials will be reviewed and approved by the Ethics Committee before use. They will clearly describe the research purpose, procedures, potential risks and benefits, voluntariness, withdrawal rights, privacy protection, and contact information, and will not contain coercive, misleading, exaggerated, or improperly inducive language.

**3.3.3 Entry Criteria**

Participants will be eligible if they meet all of the following criteria:

1) SO was diagnosed based on the concurrent presence of sarcopenia, obesity, and adequate baseline functional capacity. Sarcopenia will be defined according to the Asian Working Group for Sarcopenia (AWGS) 2019 criteria, requiring low muscle strength alongside either reduced physical performance or low muscle mass. Low muscle strength will be defined as handgrip strength <28 kg for men and <18 kg for women. Reduced physical performance or low muscle mass will be defined as a Short Physical Performance Battery (SPPB) score ≤9, or an appendicular skeletal muscle mass index (ASMI) <7.0 kg/m2 in men and <5.4 kg/m2 in women, assessed using dual-energy X-ray absorptiometry (DXA). Obesity will be defined as either a body mass index (BMI) ≥28 kg/m2 or a body fat percentage >25% in men and >35% in women, measured using the InBody 770 device.

2) Age ≥60 years;

3) Ability to safely participate in the intervention;

4) Willingness to undergo study assessments and provision of written informed consent voluntarily.

**3.3.4 Exclusion Criteria**

Participants meeting any of the following criteria will be excluded:

1) Acute myocardial infarction, unstable angina, stroke, heart failure, or other severe cardiovascular or cerebrovascular diseases that preclude participation in exercise training or physical function assessments;

2) Severe hepatic or renal insufficiency, uncontrolled severe endocrine disorders, active malignancy, or current receipt of radiotherapy;

3) Diabetes mellitus;

4) Severe psychiatric disorders, cognitive impairment, or any other condition that would impair compliance with the intervention or follow-up;

5) Severe osteoarticular disease causing marked limitation of motor function;

6) Current participation in another clinical trial.

**3.3.5 Exit/Abort Criteria**

Participants may discontinue the intervention or withdraw from the study at any time. Discontinuation of the intervention will not be regarded as withdrawal from the study unless the participant explicitly requests withdrawal or declines further follow-up. Participants who discontinue the intervention will be encouraged to complete all scheduled follow-up assessments whenever possible. The intervention may be temporarily suspended or permanently discontinued at the investigator’s discretion under any of the following circumstances:

1) occurrence of a serious adverse event, an adverse event considered related to the intervention, or any other condition that makes continued intervention unsafe;

2) significant deterioration in key health indicators or the development of clinical conditions that make continued intervention inappropriate;

3) persistent inadequate adherence despite reminders and assessment of possible causes, defined as weekly adherence of <50% for two consecutive weeks or overall adherence of <50% during the study period;

4) inability to understand or follow the intervention recommendations or a newly identified or previously undisclosed medical condition that may compromise intervention safety;

5) a request by the participant to discontinue the intervention or withdraw from the study.

For participants who discontinue the intervention or withdraw from the study, investigators will make reasonable efforts, with the participant’s consent, to document the reason for and date of discontinuation or withdrawal and to complete feasible safety and outcome assessments. Appropriate clinical management will be provided if discontinuation is related to adverse events, deterioration in health status, or unsatisfactory treatment effects.

All available data collected before discontinuation or withdrawal will be included in the analysis unless the participant withdraws consent for data use. No replacement participants will be recruited. All randomized participants will be analyzed according to the intention-to-treat (ITT) principle, regardless of intervention discontinuation, protocol deviations, or adherence level. Protocol deviations, unresolved data-quality issues, and adherence-related issues will be documented and considered in sensitivity or per-protocol analyses, as appropriate..

**3.3.6 Quitting Subject Processing**

(1) For all participants who discontinue the intervention or withdraw from observation, researchers will attempt to determine the reason for withdrawal through home visits, telephone, WeChat, or written communication, record the date of the last intervention or assessment, and document the reason in the case report form (CRF).

(2) With the participant’s consent, the research team will make every effort to complete feasible safety assessments and collect primary outcome data before withdrawal or as soon as possible thereafter.

(3) All available data from withdrawn participants will be securely retained and included in the intention-to-treat analysis according to the original group allocation. No replacement participants will be recruited for withdrawn cases.

(4) If withdrawal occurs because of an adverse event related to the study, the research team will provide or arrange necessary medical care and follow-up until the event is properly resolved or stabilized, in accordance with institutional policies and applicable regulations.

**IV. Research Process and Intervention Schemes**

**4.1 Study Total Process**

The total duration for each participant will be 48 weeks, including a 24-week intervention period followed by a 24-week follow-up period. The process includes screening (week −1 to week 0), informed consent, baseline assessment (week 0), randomization after completion of baseline assessment, intervention period (weeks 1 to 24), interim assessments (weeks 8 and 16), post-intervention assessment (week 24), and long-term follow-up assessment (week 48). After week 24, both groups will enter a follow-up phase without systematic intervention and will be observed until week 48.

Potential participants will first undergo screening to determine eligibility, including assessment of medical history and lifestyle habits. Individuals meeting the eligibility criteria will be invited to an in-person visit, during which the study procedures will be explained and written informed consent will be obtained. All baseline assessments will be completed on a single day following participant enrollment. Upon arrival at the hospital, participants will complete questionnaires collecting demographic and clinical information, including sex, age, height, weight, marital status, home address, and contact information. Body composition measurements and physical fitness tests will then be performed to assess mobility, muscle strength, and overall physical function. Participants will also complete supplementary questionnaires at home covering dietary status, quality of life, and SO-related symptoms.

After baseline assessment, trained research staff will assist participants in the intervention group with downloading and using the SO-KGCS system. Based on baseline assessment results, the system will provide personalized plans for nutrition, exercise, and lifestyle modification. Participants in the intervention group will receive regular personalized updates through the system, while those in the control group will receive standard health education. The intervention period will last 24 weeks. During the study, all participants will be instructed to maintain a daily calorie intake approximately 200 kcal below their baseline levels, in accordance with the ESPEN and EASO Consensus Statement on SO. Daily protein intake will be maintained at ≥1.2 g/kg body weight. Adherence, adverse events, and reasons for withdrawal will be recorded throughout the intervention period.

**4.2 Randomization and Blindness**

Participants will be randomized in a 1:1 ratio to the intervention or control group using a computer-generated block randomization sequence prepared by an independent statistician, using random blocks of 4 or 6 to prevent prediction of assignment. Allocation concealment will be maintained through a password-protected centralized web-based system accessible only to an independent registrar after completion of baseline assessments. Because of the nature of the personalized lifestyle intervention, participants and intervention providers cannot be blinded. However, outcome assessors and statisticians will remain blinded throughout the study. Outcome assessments will be completed by standardized independent assessors unaware of group allocation. Participants will be instructed not to disclose their group allocation to assessors. Intervention providers will not participate in outcome assessments. An emergency unblinding protocol supervised by the Ethics Committee will also be established to address potential safety risks.

**4.3 Intervention Measures**

**4.3.1 Control Group: Regular Care Group**

Participants in the control group will receive routine SO lifestyle management and health education only, without personalized recommendations generated by SO-KGCS.

Contents: After discharge or outpatient visits, each participant will receive an SO lifestyle management handbook and verbal guidance on basic SO management in older adults. The education will include risks associated with sarcopenia and obesity, dietary recommendations such as increasing protein intake and reducing high-fat and high-sugar foods, exercise advice such as daily walking and simple limb activities according to individual safety, and chronic disease management measures such as adequate sleep and regular monitoring of general health indicators when clinically indicated.

Interventions include:

Dietary aspects:

(1) All participants will be instructed to maintain a daily calorie intake approximately 200 kcal below their baseline levels, while maintaining protein intake at ≥1.2 g/kg body weight/day where clinically appropriate. Dietary protein should be distributed across meals, and high-quality protein sources such as lean meat, fish and shrimp, milk, eggs, soy products, and other appropriate foods may be recommended as general health education.

(2) Older adults should avoid extremely low-energy diets (<1,000 kcal/day) to reduce the risk of inhibiting skeletal muscle protein synthesis, accelerating muscle decomposition, decreasing muscle strength, and causing fluid or electrolyte imbalance.

(3) Vitamin D, HMB, essential amino acids, vitamin B, magnesium, calcium, and other micronutrient supplements will not be routinely prescribed as part of the control-group research intervention. Their use will be permitted only when clinically indicated and prescribed or approved by the participant’s treating physician, and all such use will be documented.

(4) Participants will receive general education to limit high glycemic index foods, control daily salt intake to ≤5 g, cooking oil intake to 25–30 g, and added sugar intake to ≤50 g (preferably <25 g), and to increase intake of vegetables, fruits, beans, tea, and other foods consistent with individual dietary safety and preferences.

Exercise aspects:

(1) Based on the principles of safety, individual capacity, and gradual progression, routine education will encourage appropriate resistance training, aerobic exercise, flexibility training, and multicomponent training where feasible.

(2) Participants will be advised to perform exercise within their safe functional capacity. Any individualized exercise prescription beyond routine education should be provided by qualified clinicians when clinically needed; the control group will not receive SO-KGCS-generated personalized exercise recommendations.

(3) Before exercise training, participants should warm up for 3 to 5 minutes through slow walking, joint movement, or other low-intensity activities to improve exercise efficiency and reduce the risk of muscle, ligament, and joint injury.

(4) Resistance exercise education may include low-intensity resistance training at approximately 40%–60% of one-repetition maximum (1RM), with gradual progression when safe and tolerated. Elastic bands, sandbags, dumbbells, or body-weight exercises may be used as examples of common resistance tools.

(5) Aerobic exercise education may include walking, leg lifting, cycling, or other suitable activities for approximately 30 to 45 minutes per session, at least three times per week when tolerated, aiming for moderate intensity within a safe heart-rate range as advised by clinical staff.

(6) Participants will be advised to choose safe exercise locations, avoid exercising alone when health risks are present, stop exercise immediately if chest pain, severe dizziness, dyspnea, palpitations, or other warning symptoms occur, and seek medical help when needed.

Psychological aspects:

(1) Mental health concerns such as depression or anxiety will be screened or discussed when clinically indicated, with referral to appropriate clinical services if needed.

(2) Cognitive behavioral therapy or other psychological treatment will not be provided as a mandatory trial intervention. If psychological treatment is clinically indicated, it will be arranged through routine medical pathways and documented as concomitant care.

**Follow-up aspects:**

(1) Participants will be advised to quit smoking and limit or avoid alcohol consumption.

(2) Participants will be advised to reduce sedentary behavior and passive screen time. They may be encouraged to stand or perform light activity for 3 to 5 minutes every hour when safe.

(3) A safe and gradual weight-management goal may be discussed as general education, but rapid or excessive weight loss will be discouraged to protect skeletal muscle mass and participant safety.

(4) Routine clinical follow-up frequency will be arranged according to clinical needs. During the study, scheduled research follow-up will be conducted according to the protocol to collect adherence, safety, and outcome data.

**4.3.2 Intervention Group: SO-KGCS Intervention Group**

Participants in the intervention group will receive the same routine lifestyle guidance as the control group and, in addition, will use the SO-KGCS mobile application to receive a 24-week personalized and dynamic lifestyle intervention.

System Overview: SO-KGCS is a mobile health platform developed specifically for patients with SO. It integrates a knowledge graph, patient personal health data, and intelligent recommendation algorithms. A systematic search was conducted across multiple databases and relevant websites, including BMJ Best Practice, UpToDate, the Joanna Briggs Institute (JBI, Australia), the Center for Evidence-Based Healthcare, the Guidelines International Network (GIN), the Cochrane Library, OVID, PubMed, Web of Science, the European Society for Clinical Nutrition and Metabolism, the Academy of Nutrition and Dietetics, the Society for Parenteral and Enteral Nutrition, MedLink, the China Biomedical Literature Service Network, CNKI, Wanfang, and VIP, to retrieve lifestyle-related content concerning SO. The search period extended from database inception to August 1, 2025. Following expert review and revision by a multidisciplinary panel of endocrinologists, nurses, and nutritionists, and considering Chinese national conditions and patient needs, the final intervention plan and knowledge graph were established. The knowledge map integrates multidimensional information such as disease characteristics, nutritional requirements, exercise modalities, TCM interventions, lifestyle factors, contraindications, precautions, and safety considerations, thereby forming a structured intervention knowledge base to support personalized recommendations.

**Implementation steps:**

(1) Installation and registration: Research assistants will assist participants in installing the app on their personal phones or an approved device, completing registration, and learning basic system operations.

(2) Entry of baseline data: Participants will enter or confirm demographic characteristics, functional indicators, physiological and biochemical parameters, medical history, medication history, lifestyle habits, dietary preferences, and baseline assessment data in the app, with assistance from research staff when necessary.

(3) Plan generation: Based on the knowledge graph and algorithmic model, the system will automatically generate a personalized comprehensive intervention plan comprising disease assessment, treatment objectives, exercise training, dietary modification, TCM-related lifestyle recommendations where appropriate, lifestyle optimization, and necessary medical recommendations or reminders, together with expected outcomes, contraindications, precautions, and safety considerations. The system is intended to support, not replace, clinician judgment.

Nutritional Prescription: Total daily calorie target will generally be approximately 200 kcal below baseline intake, with protein intake maintained at ≥1.2 g/kg body weight/day where clinically appropriate. The plan may include macronutrient distribution, meal-by-meal allocation, and specific food recommendations.

Exercise prescriptions: The plan may include exercise type, frequency, intensity, duration, specific movements, safety considerations, and video instruction, covering resistance, aerobic, and flexibility exercises as appropriate.

Lifestyle recommendations: The plan may include sleep, stress management, hydration, interruption of sedentary behavior, smoking cessation, alcohol limitation, and other lifestyle recommendations.

Chinese Medicine Recommendations: Where appropriate and safe, the system may provide non-invasive TCM-related lifestyle recommendations such as medicinal dietary suggestions or massage/acupressure education. These recommendations will not replace clinical diagnosis or physician-prescribed treatment.

(4) Execution and Recording: Participants will record diet, exercise completion, and subjective feelings such as fatigue, hunger, discomfort, or other symptoms through the app on a regular basis.

(5) Dynamic feedback and adjustment: The system will adjust or remind participants according to recorded data, weekly brief questionnaires, and preset algorithmic rules. The feedback section will allow participants to report their experiences, diet and exercise completion, fatigue, hunger, discomfort, or other symptoms. Researchers will remotely monitor system alerts, such as lack of recording for consecutive days or self-reported discomfort, and medical staff will adjust recommendations when necessary through in-app messages or telephone calls.

(6) Social support: Participants will be encouraged to include a spouse, relative, or friend in the app-based family support circle, so that the supporter can understand and facilitate lifestyle changes while respecting participant privacy and autonomy.

Concomitant treatment management: Participants may continue stable existing medications and receive necessary emergency care. Participation in other structured programs targeting weight loss, muscle gain, or physical function improvement, as well as unauthorized use of medications or supplements that may materially affect muscle metabolism or body composition, will be prohibited unless clinically indicated and approved by the investigator. All concomitant treatments and relevant events will be documented and considered in the analysis.

**4.4 Strategies to Improve Compliance**

The technical level: The app interface is designed to be simple and user-friendly, with reminders, graphic guidance, and video instruction to reduce barriers to use.

Personnel Level: The research team will provide close monitoring, regular targeted clinical guidance, biweekly follow-up visits, and supportive education through mobile applications. A designated research coordinator will maintain regular communication with intervention-group participants, provide technical support, answer questions, and offer supportive education. Additional weekly technical support may be provided during the early implementation stage when needed.

Family and Social Support: With participant consent, family members or trusted supporters may be involved at study initiation to support recommended lifestyle modifications and help maintain adherence.

Incentive Measures: Small transportation subsidies or health-related gifts of ethically appropriate value may be provided to participants who complete major assessment time points on schedule. Incentives will not be excessive and will not affect participants’ voluntary decision-making.

Additional retention strategies: The study team will reduce participant burden through optimized procedures such as telephone follow-up and online questionnaires where appropriate, maintain engagement through regular health newsletters, and establish a multi-channel contact mechanism with backup contact information to support long-term follow-up completeness.

**V. Evaluation Time Points and Research Flow Charts**


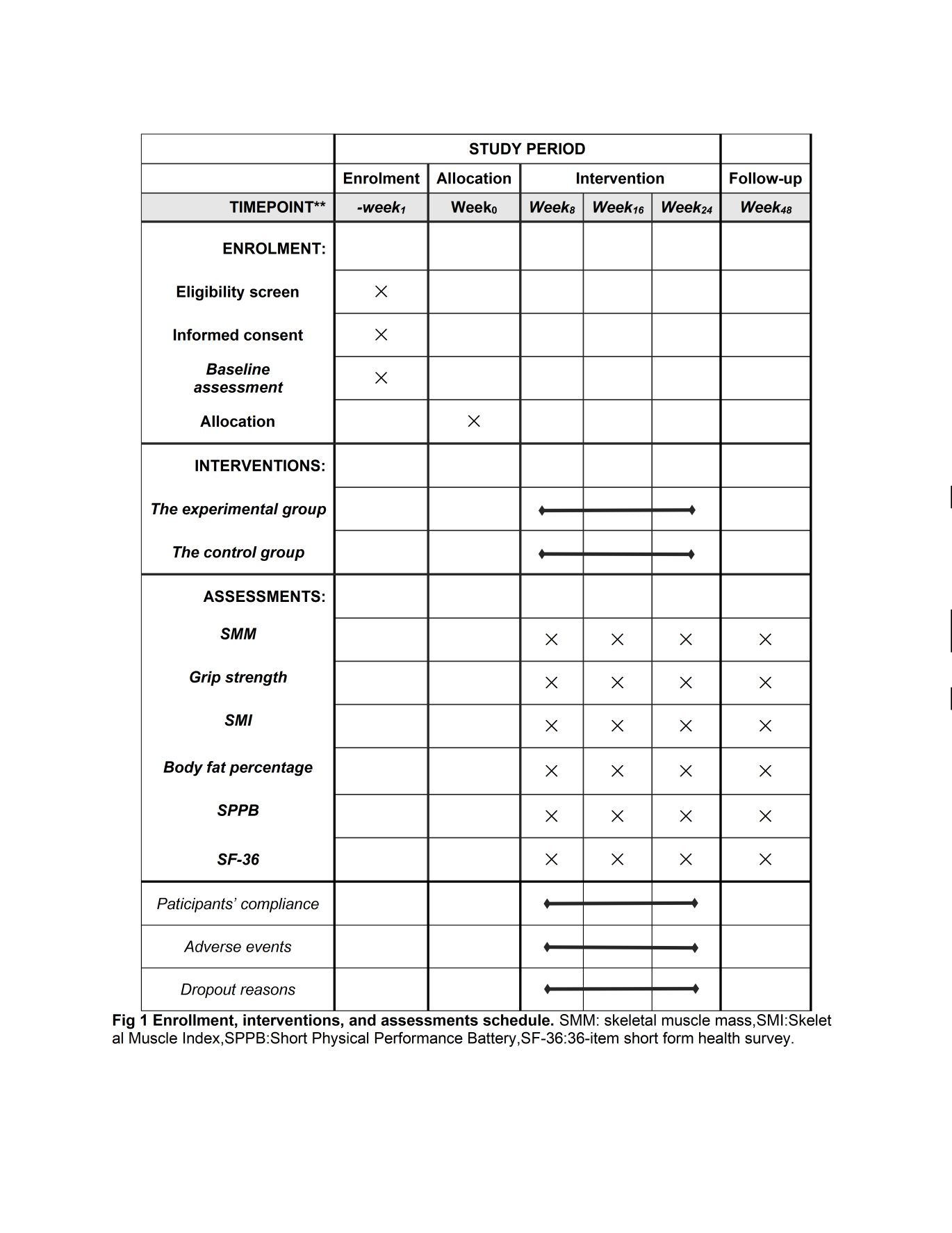


**VI. Measures**

**6.1 Primary outcome measures**

The primary outcome measure of this study is skeletal muscle mass (SMM, kg) assessed using bioelectrical impedance analysis (BIA). Before measurement, participants will be instructed to avoid strenuous exercise, alcohol consumption, and excessive fluid intake, and to empty their bladders. Measurements will be performed under resting conditions according to a standardized protocol. Participants will stand barefoot on the electrode plates of the body composition analyzer, hold the hand electrodes with both hands, maintain an upright posture, and keep the upper limbs naturally abducted without touching the trunk. All measurements will be conducted by trained researchers during the same time period and under consistent environmental conditions to minimize the influence of diet, exercise, and hydration status. SMM will be recorded in kilograms (kg) to one decimal place.

**Secondary outcome measures**

1) Grip Strength

Grip strength will be measured using a dynamometer (Jamar Plus+, USA) and recorded in kilograms. Participants will adjust the grip width to a comfortable position and perform maximal voluntary contraction for 3 seconds. Three measurements will be obtained for each hand and the highest value will be recorded. If the variation between measurements exceeds 10%, participants will rest for 10–15 minutes before repeat testing.

2) Skeletal Muscle Mass Index

Skeletal muscle mass index (SMI, kg/m²) will be assessed as a secondary outcome. Appendicular skeletal muscle mass (ASM, kg) will be measured using BIA with the same analyzer used for SMM assessment. Measurement procedures and precautions will be identical to those described for the primary outcome. SMI will be calculated as ASM divided by height squared: SMI = ASM (kg) / height² (m²). Results will be expressed in kg/m² and recorded to two decimal places.

3) Body Fat Percentage

Body fat percentage will be measured using the same body composition analyzer under standardized conditions as described above. Results will be expressed as percentages (%) and recorded to one decimal place.

4) Physical Function Assessment

Physical function will be assessed using the Short Physical Performance Battery (SPPB), which includes a balance test, a 4-meter usual gait speed test, and a five-times sit-to-stand test. These components assess balance, walking ability, lower-limb muscle strength, and endurance, respectively. The balance test includes side-by-side, semi-tandem, and tandem standing positions. Each component will be scored from 0 to 4, yielding a total score ranging from 0 to 12, with higher scores indicating better physical function.

5) Quality of Life

Quality of life will be assessed using the MOS 36-item Short Form Health Survey (SF-36). The SF-36 evaluates eight domains of health-related quality of life: Physical Functioning (PF), Role-Physical (RP), Bodily Pain (BP), General Health (GH), Vitality (VT), Social Functioning (SF), Role-Emotional (RE), and Mental Health (MH). Scores for each domain will be converted to a 0–100 scale using the following formula: Converted Score = (Actual Score − Minimum Score for that Dimension) / (Maximum Score for that Dimension − Minimum Score for that Dimension) × 100. The mean score for the eight domains will be calculated as the composite score, with higher scores indicating better quality of life.

**VII. Adverse Events and Risk Management**

**7.1 Identifying Potential Risks**

(1) Exercise-related risks: muscle strain, joint pain, falls, transient fatigue, and rare cardiovascular events.

(2) Nutrition-related risks: hunger, fatigue, dizziness, gastrointestinal discomfort, dehydration, electrolyte imbalance, or hypoglycemia-like symptoms in susceptible participants caused by inappropriate calorie restriction or insufficient intake.

(3) Technical/Psychological Risks: anxiety related to app use, concerns about data privacy, frustration because of not achieving goals, or increased burden from recording diet and exercise data.

**7.2 Risk prevention and mitigation measures**

(1) Strict screening: High-risk individuals will be excluded through eligibility assessment, medical history review, and investigator judgment before enrollment.

(2) Personalization and Progressivity: SO-KGCS-generated exercise prescriptions will begin at a low and safe intensity and increase gradually. Nutritional plans will ensure minimum safe caloric and protein intake and will avoid extreme calorie restriction.

(3) Safety Education: The app will include exercise safety tips and warm-up/relaxation tutorials. Participants will be clearly informed to stop exercise immediately and seek medical care if they experience chest pain, severe dizziness, dyspnea, palpitations, falls, or other warning symptoms.

(4) Medical supervision: Physicians are included in the research team and can provide consultation when needed. Participants with abnormal glucose-related symptoms or other clinical concerns will be advised to seek medical assessment, and relevant information will be recorded and managed according to clinical judgment.

(5) Ethical training: All researchers will receive GCP and ethics training, emphasizing participant safety, voluntariness, privacy protection, and the priority of participant rights and welfare.

**7.3 Handling and reporting of adverse events**

(1) Definitions and Records: Any adverse medical event occurring during the study period, whether related to the intervention or not, will be recorded in detail in the CRF and adverse event record table, including occurrence time, severity, duration, relationship assessment, management measures, outcome, and referral if any. Events of special interest will include falls, injuries, musculoskeletal problems, hypoglycemic episodes or symptoms, major cardiovascular events, and any other events potentially related to study procedures or interventions.

(2) Treatment Principles: The study team will immediately assess adverse events, suspend or adjust the intervention when necessary, and provide or arrange appropriate medical treatment. Adverse events will be followed until symptoms or signs resolve or stabilize. Follow-up methods may include inpatient observation, outpatient follow-up, home visits, telephone follow-up, or written communication depending on event severity.

(3) Reporting process:

Serious Adverse Events (SAEs): Events leading to death, life-threatening conditions, hospitalization or prolonged hospitalization, persistent or significant disability, disfigurement, or other important medical events must be reported by the principal investigator to the Ethics Committee of the institute and relevant departments within 24 hours after awareness, and a written report must be submitted as required by regulations.

Non-serious adverse events: Non-serious adverse events will be summarized periodically and reported in research progress reports submitted to the Ethics Committee.

**7.4 Insurance and Compensation**

This study will provide trial-related clinical trial liability insurance or institutional compensation arrangements in accordance with applicable regulations and hospital policies to cover trial-related injuries. Additional examination fees required solely for study participation will be covered by the study fund. Reasonable transportation costs incurred for participation in scheduled study assessments may be reimbursed at a fixed amount approved by the Ethics Committee. Compensation or reimbursement will not be excessive and will not constitute improper inducement.

**VIII. Data Management and Statistical Analysis**

**8.1 Data Management**

Capture and Recording: Study data will be collected using pre-designed paper case report forms (CRFs) or validated electronic data capture systems (EDCs).

Entry and verification: Two uniformly trained data personnel will independently enter data into an encrypted electronic database. Data will then be checked for completeness, accuracy, and logical consistency. Any discrepancy will be verified against the original CRFs and corrected after confirmation by authorized study personnel.

Secrecy and Storage: All data will be de-identified using unique research identification numbers instead of directly identifiable personal information. Electronic data will be stored on secure servers with password protection, access control, and firewall protection. Paper documents will be stored in locked filing cabinets in restricted-access areas. The data retention period will be at least 15 years after study completion, after which data will be securely destroyed in accordance with institutional policy and applicable regulations.

Data access permissions: Only core research team members, authorized monitors, auditors, and ethics or regulatory reviewers may access study data within the scope of their duties. All personnel with access to data will sign confidentiality agreements. Publicly reported results will use aggregate data only and will not identify any individual participant.

Quality assurance of measurements: All outcome assessments will be completed by uniformly trained independent assessors who are unaware of group allocation. Body composition measurements and physical assessments will be performed using standardized procedures; where role-specific procedures apply, body composition measurements will be performed by the same trained physician using the same equipment, and physical function assessments will be performed by uniformly trained professional nurses or assessors. Data quality checks will be conducted immediately after each assessment to minimize missingness and measurement errors.

**8.2 Statistical Analysis**

The primary efficacy analysis will be conducted according to the ITT principle, with all randomized participants analyzed in the groups to which they were originally assigned. Continuous variables will be summarized as means ± standard deviations or medians with interquartile ranges, as appropriate based on distribution, and categorical variables will be presented as frequencies and percentages. Baseline characteristics will be summarized by treatment group. Between-group comparisons at baseline will be performed using independent-samples t tests or Mann-Whitney U tests for continuous variables, and χ² tests or Fisher’s exact tests for categorical variables.

The primary analysis population will be the ITT population. A per-protocol set analysis will be conducted as a sensitivity analysis and will include participants who complete the intervention and follow-up assessments, meet prespecified adherence criteria, and have no major protocol deviations. Poor adherence will be defined as an intervention adherence rate of <50% per week for two consecutive weeks or an overall intervention adherence rate of <50% during the study period. The safety analysis population will include participants who receive at least one intervention session or complete at least one post-randomization safety assessment.

The sole primary confirmatory outcome will be SMM. The primary intervention effect will be defined as the adjusted between-group difference in change in SMM from baseline to the end of the 24-week intervention. The primary outcome will be analyzed using a linear mixed-effects model, with post-randomization SMM measurements as the dependent variable. Fixed effects will include group, time, the group-by-time interaction, and prespecified covariates, including age, sex, body weight, and baseline SMM. A participant-level random intercept will account for within-participant correlation arising from repeated measurements. Time will be modeled as a categorical variable to avoid imposing a linear change trajectory. The primary effect will be estimated from the group-by-time interaction at week 24, and the adjusted between-group mean difference, 95% confidence interval, and two-sided P value will be reported.

Secondary outcomes will include handgrip strength, skeletal muscle index, body fat percentage, SPPB score, and SF-36 quality-of-life score. Each secondary outcome will be analyzed using a linear mixed-effects model analogous to that used for the primary outcome. Fixed effects will include group, time, the group-by-time interaction, and prespecified covariates, including age, sex, body weight, and the baseline value of the corresponding outcome. Adjusted between-group differences, 95% confidence intervals, and two-sided P values will be reported. SMM will be the only primary confirmatory outcome, whereas secondary outcomes will provide supportive evidence regarding intervention effects. For analyses involving multiple secondary outcomes and repeated time points, the Benjamini–-Hochberg procedure will be applied to control the false discovery rate.

All available repeated-measures data will be included in the linear mixed-effects models using maximum likelihood estimation under the missing-at-random assumption. The extent, timing, and pattern of missing data, together with reasons for withdrawal or intervention discontinuation, will be summarized by group. For the primary outcome, a sensitivity analysis will be conducted using multiple imputation by chained equations. The imputation model will include randomized group, age, sex, height, body weight, BMI, baseline SMM, available follow-up SMM measurements, and other variables potentially associated with missingness or the primary outcome. Twenty imputed datasets will be generated and analyzed using the primary analysis model, with estimates pooled according to Rubin’s rules. If missingness in the primary outcome is substantial or the missing-data mechanism remains uncertain, a tipping-point analysis based on delta-adjusted multiple imputation will be performed. In this analysis, a series of prespecified unfavorable shifts will be applied to the imputed SMM values to assess the robustness of the primary conclusions under plausible missing-not-at-random assumptions.

Prespecified exploratory subgroup analyses will be performed by age and sex through inclusion of group-by-time-by-subgroup interaction terms in the linear mixed-effects model. Model assumptions will be evaluated using residual plots, normal Q-Q plots, and outlier diagnostics. If substantial heteroscedasticity or deviation from residual non-normality is detected, robust standard errors will be used in sensitivity analyses while retaining the primary model structure. Safety analyses will be primarily descriptive. Adverse events and serious adverse events will be summarized by group, severity, possible relationship to the intervention, and outcome. All statistical tests will be two-sided, and P<0.05 for the primary outcome will be considered statistically significant. Statistical analyses will be performed using R software, version 4.4.2 (R Foundation for Statistical Computing, Vienna, Austria).

**IX. Ethical Considerations**

**9.1 Informed Consent**

Potential participants will be given a complete and comprehensibly worded informed consent form. A trained researcher will spend sufficient time, usually no less than 30 minutes, explaining the study purpose, procedures, randomization, intervention content, potential benefits, foreseeable risks, privacy protection measures, compensation and insurance arrangements, voluntary participation, withdrawal rights, and contact information. Participants and, when appropriate, their family members will be given sufficient time to consider participation, generally at least 24 hours. Written informed consent will be obtained before any study-specific procedure. Two copies of the informed consent form will be signed, one retained by the participant and one retained by the research team.

**9.2 Benefits and Risks**

Benefits: Participants will receive free study-related body composition and physical function assessments, routine lifestyle guidance, and continued research attention. Participants in the intervention group may additionally receive personalized lifestyle recommendations through SO-KGCS, which may help improve body composition, muscle strength, physical function, and quality of life.

Risk: The foreseeable risks are mainly related to exercise, dietary adjustment, app use, and privacy concerns. These risks are expected to be manageable and low under strict screening, progressive intervention, standardized safety education, medical supervision, adverse event monitoring, and data protection measures. The control group will receive routine lifestyle education and will not be deprived of standard care.

**9.3 Privacy and confidentiality**

This study will strictly protect participants’ personal privacy and health information. All research documents, databases, and biological samples, if any, will use research identification numbers rather than directly identifiable information. Publicly published or presented research results will be aggregate data only and will never disclose information that can identify an individual participant. After study completion, de-identified data may be used for future scientific research only under the premise of compliance with applicable laws, regulations, and ethical requirements, and additional ethics approval will be sought when required.

**9.4 Vulnerable Groups**

This study does not specifically target children, pregnant women, people with cognitive disabilities, or other particularly vulnerable populations. Because eligible participants are older adults, the research team will ensure that the informed consent process is fully understandable, voluntary, and adequately communicated. When necessary and with participant permission, trusted family members may be present to assist communication, but the participant’s own wishes and autonomy will be respected.

**9.5 Communication of Study Results**

After study completion, the results will be submitted for publication in peer-reviewed academic journals and may be reported at academic conferences. The research team will provide feedback on overall, non-individualized study results to participants who are willing to receive them, using a summarized and broadly understandable format. No identifiable personal information will be disclosed in any publication, presentation, or feedback material.

**9.6 Quality Control and Guarantee**

(1) Establish an independent Research Guidance Committee or equivalent study oversight mechanism to supervise scientific validity, participant safety, protocol implementation, and overall study progress.

(2) Conduct periodic internal data checks and quality control reviews, including verification of CRFs, informed consent documents, eligibility documentation, intervention records, adverse event records, and outcome assessment completeness.

(3) Accept continuing review, progress review, and inspection by the Ethics Committee of the institute, and promptly submit safety reports, protocol amendments, and study progress reports as required.

**研究方案**

**研究项目名称：基于知识图谱的生活方式干预推荐系统在改善肌少性肥胖患者身体成分、肌肉力量和躯体功能方面的有效性：一项随机对照试验研究方案**

**项目来源：广州中医药大学“双一流”学科高质量发展“筑峰造尖”行动计划（2024—2027年）（GZY2025GB0925）；深圳市福田区卫生系统科研项目（FTWS2025038）；国家卫生健康委能力建设和继续教育中心慢病管理研究项目（GWJJMB202510024225）。**

**项目负责人：陈赟**

**版本：V2.0**

**版本日期：2026年4月28日**

**研究者声明**

作为本研究项目的主要研究者，我将遵守国家卫生和计划生育委员会（2016）发布的涉及人的生物医学研究伦理审查办法、世界医学会《赫尔辛基宣言》（2013）、CIOMS《涉及人的生物医学研究国际伦理准则》以及GCP伦理原则。我将在临床试验质量管理规范的指导下，按照医学伦理委员会批准的研究方案开展本研究，以确保研究的科学性，并维护受试者的健康和权益。

姓名：陈赟

日期：2026年4月28日

**一、研究背景**

肌少性肥胖（sarcopenic obesity，SO）的特征是肌少症与肥胖并存，并被认为是一种独立的临床状态，而不是两种疾病的简单叠加。在SO中，过量脂肪堆积与骨骼肌量减少、肌力受损和身体功能下降相互作用，增加代谢异常、衰弱、残疾、跌倒、心血管疾病以及生活质量下降的风险。因此，SO给患者、家庭、医疗服务体系和社会带来沉重负担。

生活方式干预仍是SO管理的重要策略。有效干预需要同时改善肌肉量和肌力，并减少过量脂肪。然而，SO患者在年龄、身体功能、合并症、代谢状态、饮食习惯、运动能力和行为偏好方面存在显著差异。这些差异使标准化或人工制定的生活方式建议难以在常规临床实践中实现个体化、执行和长期维持。

基于知识图谱的推荐系统能够整合结构化医学知识和多维患者数据，从而为营养、运动、生活方式调整以及非侵入性中医健康管理提供个体化建议。本研究将评价肌少性肥胖知识图谱照护系统（Sarcopenic Obesity-Knowledge Graph Care System，SO-KGCS）在改善老年SO患者骨骼肌量及相关结局方面的有效性和安全性。

**二、研究目的与意义**

**2.1 主要目的**

评价在常规生活方式指导基础上，通过SO-KGCS实施24周个体化生活方式干预，与单纯常规生活方式指导相比，是否能够改善SO患者骨骼肌量（SMM）。

**2.2 次要目的**

评价SO-KGCS对握力、骨骼肌指数（SMI）、体脂率、身体功能和健康相关生活质量的影响。

通过监测跌倒、损伤、肌肉骨骼症状、低血糖事件、主要心血管事件及其他不良事件，评价SO-KGCS干预的安全性。

评估干预依从性、系统可用性以及将基于知识图谱的推荐系统整合到SO管理中的可行性。

探索干预反应是否因预设的受试者特征（如年龄和性别）而不同。这些分析为探索性分析，不作为主要疗效结论的判定依据。

**2.3 研究意义**

本研究将提供临床证据，说明智能生活方式推荐系统能否支持SO精准、可行且安全的慢性病管理。若证实有效，SO-KGCS可能降低多学科团队的工作负担，并提高老年SO患者获得个体化生活方式干预的可及性。

**三、研究方法**

**3.1 研究设计**

本研究为单盲、双臂随机对照试验，旨在评价基于知识图谱的生活方式推荐系统对SO患者骨骼肌量、握力、骨骼肌指数、体脂率、身体功能和生活质量的影响。受试者将按1:1比例随机分配至干预组或对照组。干预组将接受为期24周、旨在增加肌肉量并降低体重的生活方式干预；对照组将接受常规治疗和健康教育，不接受系统生成的个体化推荐。本研究总持续时间为48周，包括24周干预期和随后24周随访期。评估将在基线以及第8、16、24和48周进行，其中第24周为干预结束时间点。此后，两组均进入无系统干预的随访阶段，并观察至第48周。随机序列将由统计学家采用计算机生成的区组随机法产生，区组大小为4或6。基线评估完成后，将通过受密码保护的集中网络系统进行分配隐藏，该系统仅由不参与结局评估的独立登记员访问。由于生活方式干预的特点，无法对受试者和干预实施者设盲。然而，结局评估者和统计分析人员将在整个研究过程中保持盲法。所有结局评估将由经过统一培训且不知晓分组情况的独立评估者完成。数据管理员和统计分析人员将在数据库锁定前保持盲态。将告知受试者不要向评估者透露其分组情况；如有需要，将建立紧急揭盲程序。

干预报告将遵循干预描述与复制模板（Template for Intervention Description and Replication，TIDieR）清单，研究方案的制定符合SPIRIT声明。拟定的研究时间安排和程序见入组、干预、评估时间表及研究流程图。

**3.2 研究场所**

受试者将通过广州中医药大学深圳医院（福田）内分泌科招募并评估，必要时由合作的基层医疗卫生机构提供支持。

**3.3 研究对象**

**3.3.1 样本量计算**

样本量采用G-power 3.1软件计算。计算基于两组骨骼肌量（SMM）平均变化量的双侧比较，并将SMM预先设定为唯一主要确证性结局。预期效应量来源于既往针对肌少症老年人的互联网营养与运动干预研究。该研究中，对照组SMM平均变化量为−0.10 kg，综合干预组为1.09 kg，组间差异为1.19 kg。由于该研究未直接报告SMM变化量的标准差，本研究根据其报告的基线和干预后标准差进行保守估计，并假设前后测相关系数为0.50。由此得到合并标准差约为2.70 kg，Cohen’s d约为0.44。尽管拟实施的干预纳入了基于知识图谱的推荐系统，可能增强干预个体化程度，但为避免高估干预效果，本研究采用该中等效应量。在双侧α=0.05、把握度为0.80、分配比例为1:1的条件下，每组需83例。考虑预计20%的脱落率，每组拟纳入104例，共计208例受试者。

**3.3.2 招募策略**

住院和门诊招募：将在研究中心门诊和住院区域张贴招募海报。内分泌科医师及经过培训的医务人员将在常规临床诊疗过程中识别并转介可能符合条件的受试者。

社区招募：研究团队将通过与社区健康服务中心合作，向潜在受试者发放宣传资料，并举办说明会或健康教育活动。

线上招募：在符合医院和伦理委员会要求的前提下，可在机构授权的社交媒体平台（包括微信、抖音和小红书）发布招募公告。

所有招募材料在使用前均须经伦理委员会审查批准。材料将清晰说明研究目的、程序、潜在风险与获益、自愿性、退出权利、隐私保护和联系方式，不含强制性、误导性、夸大性或不当诱导性语言。

**3.3.3 纳入标准**

受试者需同时符合以下所有标准方可纳入：

1）SO的诊断基于肌少症、肥胖以及足够基线功能能力同时存在。肌少症将依据亚洲肌少症工作组（Asian Working Group for Sarcopenia，AWGS）2019标准定义，即低肌力并伴有身体功能下降或肌肉量降低。低肌力定义为男性握力<28 kg、女性<18 kg。身体功能下降或肌肉量降低定义为简易体能状况量表（Short Physical Performance Battery，SPPB）评分≤9，或采用双能X线吸收测定法（DXA）评估的四肢骨骼肌量指数（ASMI）男性<7.0 kg/m²、女性<5.4 kg/m²。肥胖定义为体重指数（BMI）≥28 kg/m²，或采用InBody 770设备测量的体脂率男性>25%、女性>35%。

2）年龄≥60岁；

3）能够安全参与干预；

4）愿意接受研究评估，并自愿签署书面知情同意书。

**3.3.4 排除标准**

符合以下任一标准者将被排除：

1）急性心肌梗死、不稳定型心绞痛、脑卒中、心力衰竭，或其他妨碍参加运动训练或身体功能评估的严重心脑血管疾病；

2）严重肝功能或肾功能不全、未控制的严重内分泌疾病、活动性恶性肿瘤，或当前正在接受放射治疗；

3）糖尿病；

4）严重精神障碍、认知功能障碍，或其他会影响干预或随访依从性的情况；

5）导致运动功能明显受限的严重骨关节疾病；

6）当前正在参加其他临床试验。

**3.3.5 退出/终止标准**

受试者可在任何时间停止干预或退出研究。除非受试者明确要求退出或拒绝进一步随访，停止干预不视为退出研究。停止干预的受试者将被鼓励尽可能完成所有计划随访评估。研究者可根据判断，在以下任一情况下暂时暂停或永久终止干预：

1）发生严重不良事件、被认为与干预相关的不良事件，或任何使继续干预不安全的其他情况；

2）关键健康指标显著恶化，或出现使继续干预不适宜的临床情况；

3）尽管已进行提醒并评估可能原因，仍持续依从性不足，定义为连续两周每周依从性<50%，或研究期间总体依从性<50%；

4）无法理解或遵循干预建议，或新发现/此前未披露的可能影响干预安全性的医学情况；

5）受试者要求停止干预或退出研究。

对于停止干预或退出研究的受试者，在其同意的前提下，研究者将合理努力记录停止或退出的原因及日期，并完成可行的安全性和结局评估。若停止干预与不良事件、健康状况恶化或治疗效果不满意有关，将提供适当的临床处理。

除非受试者撤回数据使用同意，停止干预或退出前已收集的所有可用数据均将纳入分析。不再补招受试者。所有随机化受试者将按照意向性治疗（intention-to-treat，ITT）原则进行分析，不受干预停止、方案偏离或依从性水平影响。方案偏离、未解决的数据质量问题及依从性相关问题将予以记录，并视情况在敏感性分析或符合方案分析中考虑。

**3.3.6 退出受试者处理**

（1）对于所有停止干预或退出观察的受试者，研究者将通过家访、电话、微信或书面通信等方式尽量确定退出原因，记录最后一次干预或评估日期，并将原因记录于病例报告表（CRF）中。

（2）在受试者同意的前提下，研究团队将尽最大努力在退出前或退出后尽快完成可行的安全性评估并收集主要结局数据。

（3）退出受试者的所有可用数据将被安全保存，并按原随机分组纳入意向性治疗分析。退出病例不予补招。

（4）若因与研究相关的不良事件而退出，研究团队将依据机构政策和适用法规，提供或安排必要的医疗照护和随访，直至该事件得到妥善解决或稳定。

**四、研究流程与干预方案**

**4.1 研究总体流程**

每位受试者总研究周期为48周，包括24周干预期和随后24周随访期。流程包括筛查（第−1周至第0周）、知情同意、基线评估（第0周）、基线评估完成后的随机分组、干预期（第1至24周）、中期评估（第8和16周）、干预后评估（第24周）以及长期随访评估（第48周）。第24周后，两组均进入无系统干预的随访阶段，并观察至第48周。

潜在受试者首先接受筛查以确定资格，包括病史和生活习惯评估。符合资格标准者将被邀请参加现场访视，研究人员将说明研究程序并取得书面知情同意。受试者入组后，所有基线评估将在同一天完成。受试者到达医院后，将填写问卷以收集人口学和临床信息，包括性别、年龄、身高、体重、婚姻状况、家庭住址和联系方式。随后进行身体成分测量和体能测试，以评估行动能力、肌肉力量和整体身体功能。受试者还将在家完成补充问卷，内容包括饮食状况、生活质量和SO相关症状。

基线评估完成后，受过培训的研究人员将协助干预组受试者下载并使用SO-KGCS系统。系统将根据基线评估结果提供个体化营养、运动和生活方式调整方案。干预组受试者将通过系统定期获得个体化更新；对照组受试者将接受标准健康教育。干预期为24周。研究期间，所有受试者将按照ESPEN和EASO关于SO的共识声明，被要求每日能量摄入较基线水平约减少200 kcal。每日蛋白质摄入将维持在≥1.2 g/kg体重。干预期间将全程记录依从性、不良事件和退出原因。

**4.2 随机化与盲法**

受试者将使用由独立统计学家准备的计算机生成区组随机序列，以1:1比例随机分配至干预组或对照组；区组大小采用4或6的随机区组，以防止分组预测。基线评估完成后，将通过受密码保护的集中网络系统保持分配隐藏，该系统仅由独立登记员访问。由于个体化生活方式干预的性质，受试者和干预提供者无法设盲。然而，结局评估者和统计人员将在整个研究期间保持盲态。结局评估将由标准化培训、且不知晓分组情况的独立评估者完成。将告知受试者不得向评估者透露分组情况。干预提供者不参与结局评估。还将建立由伦理委员会监督的紧急揭盲方案，以应对潜在安全风险。

**4.3 干预措施**

**4.3.1 对照组：常规照护组**

对照组受试者仅接受常规SO生活方式管理和健康教育，不接受由SO-KGCS生成的个体化推荐。

内容：出院或门诊就诊后，每位受试者将获得SO生活方式管理手册，并接受关于老年人SO基本管理的口头指导。教育内容包括肌少症和肥胖相关风险；饮食建议，如增加蛋白质摄入、减少高脂和高糖食物；根据个体安全情况进行每日步行和简单肢体活动等运动建议；以及在临床需要时采取充足睡眠、定期监测一般健康指标等慢性病管理措施。

干预内容包括：

饮食方面：

（1）所有受试者将被要求每日能量摄入较基线水平约减少200 kcal；在临床适宜情况下，蛋白质摄入维持在≥1.2 g/kg体重/日。膳食蛋白质应分布在各餐中，并可将瘦肉、鱼虾、奶类、蛋类、豆制品及其他适宜食物等优质蛋白来源作为一般健康教育推荐。

（2）老年人应避免极低能量饮食（<1,000 kcal/日），以降低抑制骨骼肌蛋白合成、加速肌肉分解、降低肌力以及导致液体或电解质失衡的风险。

（3）维生素D、HMB、必需氨基酸、维生素B、镁、钙及其他微量营养素补充剂不作为对照组研究干预的一部分常规开具。仅在有临床指征并由受试者主管医师处方或批准时允许使用，所有此类使用均将记录。

（4）受试者将接受一般教育：限制高血糖生成指数食物，控制每日食盐摄入≤5 g、烹调用油25–30 g、添加糖摄入≤50 g（最好<25 g），并在符合个人饮食安全和偏好的前提下增加蔬菜、水果、豆类、茶及其他食物摄入。

运动方面：

（1）基于安全、个体能力和循序渐进原则，常规教育将鼓励在可行情况下进行适当的抗阻训练、有氧运动、柔韧性训练和多组分训练。

（2）将建议受试者在其安全功能能力范围内进行运动。超出常规教育的任何个体化运动处方，应在临床需要时由合格临床人员提供；对照组不接受SO-KGCS生成的个体化运动推荐。

（3）运动训练前，受试者应通过慢走、关节活动或其他低强度活动进行3至5分钟热身，以提高运动效率并降低肌肉、韧带和关节损伤风险。

（4）抗阻运动教育可包括约为一次重复最大力量（1RM）40%–60%的低强度抗阻训练，并在安全且可耐受时逐渐进阶。可将弹力带、沙袋、哑铃或自重训练作为常见抗阻工具示例。

（5）有氧运动教育可包括步行、抬腿、骑车或其他适宜活动；在可耐受时，每次约30至45分钟，每周至少3次，目标是在临床人员建议的安全心率范围内达到中等强度。

（6）将建议受试者选择安全的运动场所；在存在健康风险时避免独自运动；如出现胸痛、严重头晕、呼吸困难、心悸或其他警示症状，应立即停止运动，并在需要时寻求医疗帮助。

心理方面：

（1）在有临床指征时，将筛查或讨论抑郁、焦虑等心理健康问题，并在需要时转介至适当的临床服务。

（2）认知行为治疗或其他心理治疗不作为强制性试验干预提供。如有心理治疗临床指征，将通过常规医疗途径安排，并记录为合并照护。

**随访方面：**

（1）将建议受试者戒烟并限制或避免饮酒。

（2）将建议受试者减少久坐行为和被动屏幕时间。在安全情况下，可鼓励其每小时站立或进行3至5分钟轻体力活动。

（3）可将安全、渐进的体重管理目标作为一般教育进行讨论，但不鼓励快速或过度减重，以保护骨骼肌量和受试者安全。

（4）常规临床随访频率将根据临床需要安排。研究期间，将按照方案进行计划性研究随访，以收集依从性、安全性和结局数据。

**4.3.2 干预组：SO-KGCS干预组**

干预组受试者将接受与对照组相同的常规生活方式指导；此外，将使用SO-KGCS移动应用程序接受为期24周的个体化、动态生活方式干预。

系统概述：SO-KGCS是专为SO患者开发的移动健康平台，整合知识图谱、患者个人健康数据和智能推荐算法。研究团队系统检索了多个数据库和相关网站，包括BMJ Best Practice、UpToDate、Joanna Briggs Institute（JBI，澳大利亚）、循证卫生保健中心、国际指南网络（GIN）、Cochrane Library、OVID、PubMed、Web of Science、欧洲临床营养与代谢学会、美国营养与饮食学会、肠外与肠内营养学会、MedLink、中国生物医学文献服务系统、CNKI、万方和维普，以获取SO相关生活方式内容。检索时限为各数据库建库至2025年8月1日。经由内分泌医师、护士和营养师组成的多学科专家组评审和修订，并结合中国国情和患者需求，最终形成干预方案和知识图谱。知识图谱整合疾病特征、营养需求、运动方式、中医干预、生活方式因素、禁忌证、注意事项和安全考虑等多维信息，构建结构化干预知识库，以支持个体化推荐。

**实施步骤：**

（1）安装与注册：研究助理将协助受试者在其个人手机或经批准的设备上安装应用程序、完成注册，并学习系统基本操作。

（2）基线数据录入：受试者将在应用程序中录入或确认人口学特征、功能指标、生理生化参数、病史、用药史、生活习惯、饮食偏好和基线评估数据；必要时由研究人员协助。

（3）方案生成：基于知识图谱和算法模型，系统将自动生成个体化综合干预方案，包括疾病评估、治疗目标、运动训练、饮食调整、适宜情况下的中医相关生活方式建议、生活方式优化以及必要的医疗建议或提醒，并同时给出预期结果、禁忌证、注意事项和安全考虑。该系统旨在支持而非替代临床医师判断。

营养处方：每日总能量目标通常较基线摄入约减少200 kcal；在临床适宜情况下，蛋白质摄入维持在≥1.2 g/kg体重/日。方案可包括宏量营养素分配、分餐安排以及具体食物推荐。

运动处方：方案可包括运动类型、频率、强度、时长、具体动作、安全考虑和视频指导，并视情况涵盖抗阻、有氧和柔韧性运动。

生活方式建议：方案可包括睡眠、压力管理、饮水、打断久坐行为、戒烟、限酒及其他生活方式建议。

中医建议：在适宜且安全的情况下，系统可提供药膳建议或按摩/穴位按压教育等非侵入性中医相关生活方式建议。这些建议不替代临床诊断或医师处方治疗。

（4）执行与记录：受试者将通过应用程序定期记录饮食、运动完成情况以及疲劳、饥饿、不适或其他症状等主观感受。

（5）动态反馈与调整：系统将根据记录数据、每周简短问卷和预设算法规则进行调整或提醒。反馈模块允许受试者报告其体验、饮食和运动完成情况、疲劳、饥饿、不适或其他症状。研究人员将远程监测系统预警，例如连续多日未记录或自述不适；必要时，医务人员将通过应用程序消息或电话调整建议。

（6）社会支持：将鼓励受试者在应用程序中的家庭支持圈中纳入配偶、亲属或朋友，使支持者在尊重受试者隐私和自主性的前提下了解并促进生活方式改变。

合并治疗管理：受试者可继续使用稳定的既有药物，并接受必要的急救治疗。除非有临床指征并经研究者批准，否则禁止参加其他以减重、增肌或改善身体功能为目标的结构化项目，以及未经授权使用可能显著影响肌肉代谢或身体成分的药物或补充剂。所有合并治疗及相关事件均将记录，并在分析中考虑。

**4.4 提高依从性的策略**

技术层面：应用程序界面设计简洁、易于使用，并配备提醒、图文指导和视频指导，以降低使用障碍。

人员层面：研究团队将通过移动应用程序提供密切监测、定期针对性临床指导、每两周随访和支持性教育。指定研究协调员将与干预组受试者保持定期沟通，提供技术支持、解答问题并开展支持性教育。在早期实施阶段，如有需要，可额外提供每周技术支持。

家庭和社会支持：经受试者同意，可在研究启动时纳入家庭成员或可信任支持者，以支持推荐的生活方式改变并帮助维持依从性。

激励措施：对按时完成主要评估时间点的受试者，可提供符合伦理要求、价值适当的小额交通补贴或健康相关礼品。激励不会过度，也不会影响受试者的自愿决策。

其他保留策略：研究团队将通过优化程序减少受试者负担，例如在适当情况下采用电话随访和在线问卷；通过定期健康通讯维持参与度；并建立包含备用联系方式的多渠道联系机制，以支持长期随访完整性。

**五、评价时间点和研究流程图**


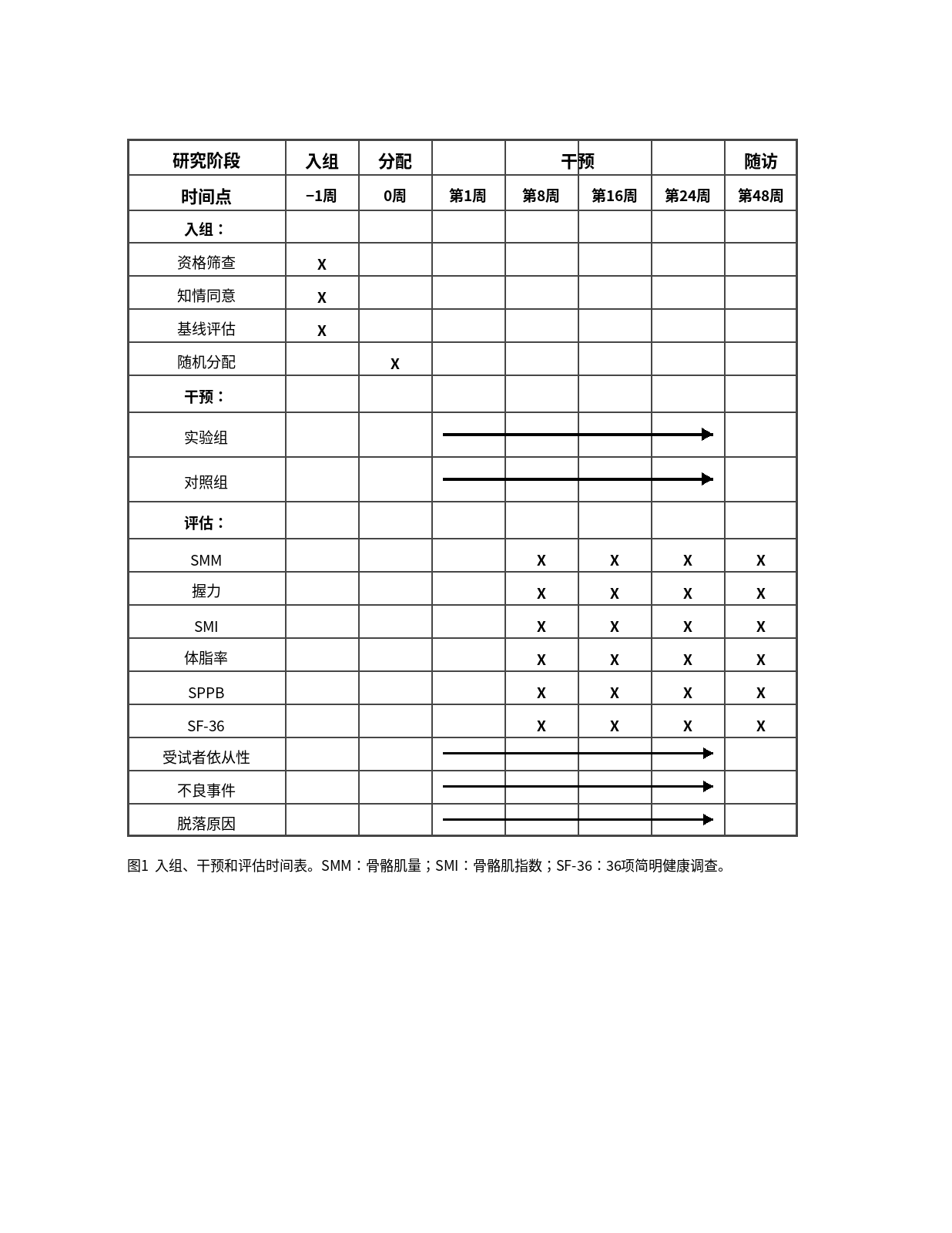


**六、测量指标**

**6.1 主要结局指标**

本研究的主要结局指标为采用生物电阻抗分析（BIA）评估的骨骼肌量（SMM，kg）。测量前，将告知受试者避免剧烈运动、饮酒和过量饮水，并排空膀胱。测量将在静息状态下按照标准化流程进行。受试者赤足站在身体成分分析仪电极板上，双手握住手电极，保持直立姿势，并使上肢自然外展、避免接触躯干。所有测量均由受过培训的研究人员在同一时间段和一致环境条件下进行，以尽量减少饮食、运动和水合状态的影响。SMM将以千克（kg）为单位记录，精确至一位小数。

**次要结局指标**

1）握力

握力将使用握力计（Jamar Plus+，美国）测量，并以千克记录。受试者将把握距调整至舒适位置，并进行3秒最大自主收缩。每只手测量3次，记录最高值。如测量间差异超过10%，受试者将休息10–15分钟后重复测试。

2）骨骼肌指数

骨骼肌指数（SMI，kg/m²）将作为次要结局进行评估。四肢骨骼肌量（ASM，kg）将采用与SMM评估相同的BIA分析仪测量。测量程序和注意事项与主要结局所述相同。SMI按ASM除以身高平方计算：SMI = ASM（kg）/身高²（m²）。结果以kg/m²表示，并记录至两位小数。

3）体脂率

体脂率将采用同一身体成分分析仪在上述标准化条件下测量。结果以百分比（%）表示，并记录至一位小数。

4）身体功能评估

身体功能将采用简易体能状况量表（SPPB）评估，该量表包括平衡测试、4米通常步速测试和五次坐站测试。这些组成部分分别评估平衡能力、步行能力、下肢肌力和耐力。平衡测试包括双脚并排站立、半串联站立和串联站立。每个组成部分评分0至4分，总分范围0至12分，分数越高表示身体功能越好。

5）生活质量

生活质量将采用MOS 36项简明健康调查量表（SF-36）评估。SF-36评价健康相关生活质量的八个维度：生理功能（PF）、生理职能（RP）、躯体疼痛（BP）、总体健康（GH）、活力（VT）、社会功能（SF）、情感职能（RE）和心理健康（MH）。各维度得分将使用以下公式转换为0–100分：转换得分=（实际得分−该维度最低分）/（该维度最高分−该维度最低分）×100。八个维度的平均分将作为综合得分，分数越高表示生活质量越好。

**七、不良事件和风险管理**

**7.1 潜在风险识别**

（1）运动相关风险：肌肉拉伤、关节疼痛、跌倒、一过性疲劳以及罕见心血管事件。

（2）营养相关风险：因能量限制不当或摄入不足而在易感受试者中引起饥饿、疲劳、头晕、胃肠不适、脱水、电解质紊乱或类似低血糖症状。

（3）技术/心理风险：与应用程序使用相关的焦虑、对数据隐私的担忧、因未达到目标而产生挫败感，或记录饮食和运动数据带来的负担增加。

**7.2 风险预防和缓解措施**

（1）严格筛查：入组前将通过资格评估、病史审查和研究者判断排除高风险个体。

（2）个体化与循序渐进：SO-KGCS生成的运动处方将从低强度且安全的水平开始，并逐步增加。营养方案将确保最低安全能量和蛋白质摄入，并避免极端能量限制。

（3）安全教育：应用程序将包含运动安全提示和热身/放松教程。将明确告知受试者，如出现胸痛、严重头晕、呼吸困难、心悸、跌倒或其他警示症状，应立即停止运动并寻求医疗照护。

（4）医疗监督：研究团队包括医师，可在需要时提供咨询。出现糖代谢相关异常症状或其他临床疑虑的受试者将被建议接受医疗评估，相关信息将根据临床判断记录并管理。

（5）伦理培训：所有研究人员均将接受GCP和伦理培训，强调受试者安全、自愿性、隐私保护以及受试者权利和福祉优先。

**7.3 不良事件的处理和报告**

（1）定义与记录：研究期间发生的任何不良医学事件，无论是否与干预相关，均将在CRF和不良事件记录表中详细记录，包括发生时间、严重程度、持续时间、相关性评估、处理措施、结局以及是否转诊。特别关注事件包括跌倒、损伤、肌肉骨骼问题、低血糖事件或症状、主要心血管事件，以及任何可能与研究程序或干预相关的其他事件。

（2）处理原则：研究团队将立即评估不良事件，必要时暂停或调整干预，并提供或安排适当医疗治疗。不良事件将随访至症状或体征消失或稳定。根据事件严重程度，随访方式可包括住院观察、门诊随访、家访、电话随访或书面通信。

（3）报告流程：

严重不良事件（SAEs）：导致死亡、危及生命、住院或住院时间延长、持续或显著残疾、毁容，或其他重要医学事件的情况，主要研究者须在知悉后24小时内向本机构伦理委员会和相关部门报告，并按法规要求提交书面报告。

非严重不良事件：非严重不良事件将定期汇总，并在提交伦理委员会的研究进展报告中报告。

**7.4 保险与补偿**

本研究将依据适用法规和医院政策提供与试验相关的临床试验责任保险或机构补偿安排，以覆盖试验相关损伤。仅因参与研究而需要的额外检查费用将由研究经费承担。参加计划研究评估所产生的合理交通费用，可按照伦理委员会批准的固定金额报销。补偿或报销不会过高，也不会构成不当诱导。

**八、数据管理与统计分析**

**8.1 数据管理**

采集与记录：研究数据将采用预先设计的纸质病例报告表（CRF）或经验证的电子数据采集系统（EDC）收集。

录入与核查：两名经过统一培训的数据人员将独立将数据录入加密电子数据库。随后将检查数据的完整性、准确性和逻辑一致性。任何差异均将与原始CRF核对，并经授权研究人员确认后更正。

保密与存储：所有数据将使用唯一研究识别号进行去标识化处理，替代可直接识别个人身份的信息。电子数据将存储在安全服务器上，并采用密码保护、访问控制和防火墙保护。纸质文件将存放于限制进入区域的上锁文件柜中。数据保存期限至少为研究完成后15年；此后将根据机构政策和适用法规安全销毁。

数据访问权限：仅核心研究团队成员、授权监查员、稽查员以及伦理或监管审查人员可在其职责范围内访问研究数据。所有可访问数据的人员均需签署保密协议。公开报告的结果仅使用汇总数据，不会识别任何个体受试者。

测量质量保证：所有结局评估将由经过统一培训、且不知晓分组情况的独立评估者完成。身体成分测量和体能评估将采用标准化程序；在适用角色分工的情况下，身体成分测量将由同一名受过培训的医师使用同一设备完成，身体功能评估将由经过统一培训的专业护士或评估者完成。每次评估后将立即进行数据质量检查，以尽量减少缺失和测量误差。

**8.2 统计分析**

主要疗效分析将按照ITT原则进行，所有随机化受试者均按其最初分配组别进行分析。连续变量将根据分布情况以均数±标准差或中位数（四分位数间距）表示，分类变量将以频数和百分比表示。基线特征将按治疗组汇总。基线组间比较中，连续变量采用独立样本t检验或Mann-Whitney U检验，分类变量采用χ²检验或Fisher确切检验。

主要分析人群为ITT人群。符合方案集分析将作为敏感性分析，纳入完成干预和随访评估、满足预设依从性标准且无重大方案偏离的受试者。依从性差定义为连续两周每周干预依从率<50%，或研究期间总体干预依从率<50%。安全性分析人群将包括至少接受一次干预或完成至少一次随机化后安全性评估的受试者。

唯一主要确证性结局为SMM。主要干预效应定义为从基线至24周干预结束时SMM变化量的调整后组间差异。主要结局将采用线性混合效应模型分析，以随机化后SMM测量值作为因变量。固定效应包括组别、时间、组别与时间交互作用，以及预设协变量（包括年龄、性别、体重和基线SMM）。受试者层面的随机截距将用于处理重复测量导致的个体内相关性。时间将作为分类变量建模，以避免强加线性变化轨迹。主要效应将依据第24周的组别与时间交互项估计，并报告调整后组间均数差、95%置信区间和双侧P值。

次要结局包括握力、骨骼肌指数、体脂率、SPPB评分和SF-36生活质量评分。每个次要结局将采用与主要结局类似的线性混合效应模型分析。固定效应包括组别、时间、组别与时间交互作用，以及预设协变量（包括年龄、性别、体重和相应结局的基线值）。将报告调整后组间差异、95%置信区间和双侧P值。SMM为唯一主要确证性结局，次要结局将为干预效果提供支持性证据。对于涉及多个次要结局和重复时间点的分析，将采用Benjamini–Hochberg程序控制错误发现率。

在线性混合效应模型中，将在数据满足随机缺失假设下采用最大似然估计纳入所有可用重复测量数据。将按组汇总缺失数据的程度、时间和模式，以及退出或停止干预的原因。对于主要结局，将采用链式方程多重插补进行敏感性分析。插补模型将包括随机分组、年龄、性别、身高、体重、BMI、基线SMM、可用随访SMM测量值以及其他可能与缺失或主要结局相关的变量。将生成20个插补数据集，并使用主要分析模型进行分析，再按Rubin法则合并估计值。若主要结局缺失较多或缺失数据机制仍不确定，将基于delta调整多重插补进行临界点分析。在该分析中，将对插补的SMM值施加一系列预设不利偏移，以评估在合理非随机缺失假设下主要结论的稳健性。

预设探索性亚组分析将按年龄和性别进行，并在线性混合效应模型中加入组别×时间×亚组交互项。模型假设将通过残差图、正态Q-Q图和离群值诊断进行评价。若检测到显著异方差或残差非正态偏离，将在保留主要模型结构的同时，在敏感性分析中使用稳健标准误。安全性分析以描述性为主。不良事件和严重不良事件将按组别、严重程度、与干预的可能关系及结局汇总。所有统计检验均为双侧，主要结局P<0.05视为具有统计学意义。统计分析将使用R软件4.4.2版（R Foundation for Statistical Computing，奥地利维也纳）进行。

**九、伦理考虑**

**9.1 知情同意**

潜在受试者将获得内容完整且表述易于理解的知情同意书。经过培训的研究人员将花费足够时间（通常不少于30分钟）说明研究目的、程序、随机化、干预内容、潜在获益、可预见风险、隐私保护措施、补偿和保险安排、自愿参与、退出权利以及联系方式。受试者及适当情况下其家庭成员将有充分时间考虑是否参加，一般不少于24小时。任何研究特异性程序开始前须取得书面知情同意。知情同意书一式两份签署，一份由受试者保留，一份由研究团队保留。

**9.2 获益与风险**

获益：受试者将免费接受研究相关身体成分和身体功能评估、常规生活方式指导以及持续研究关注。干预组受试者还可通过SO-KGCS获得个体化生活方式建议，这可能有助于改善身体成分、肌肉力量、身体功能和生活质量。

风险：可预见风险主要与运动、饮食调整、应用程序使用和隐私担忧有关。在严格筛查、渐进干预、标准化安全教育、医疗监督、不良事件监测和数据保护措施下，这些风险预计可控且较低。对照组将接受常规生活方式教育，不会被剥夺标准照护。

**9.3 隐私与保密**

本研究将严格保护受试者个人隐私和健康信息。所有研究文件、数据库以及生物样本（如有）均将使用研究识别号，而非可直接识别身份的信息。公开发表或展示的研究结果仅为汇总数据，绝不披露可识别任何个体受试者的信息。研究完成后，去标识化数据仅可在符合适用法律、法规和伦理要求的前提下用于未来科学研究；必要时将另行申请伦理批准。

**9.4 弱势群体**

本研究不专门针对儿童、孕妇、认知障碍者或其他特别弱势人群。由于符合条件的受试者为老年人，研究团队将确保知情同意过程充分易懂、自愿且沟通充分。必要时并经受试者许可，可信任家庭成员可在场协助沟通，但将尊重受试者本人的意愿和自主权。

**9.5 研究结果沟通**

研究完成后，结果将提交至同行评议学术期刊发表，并可能在学术会议上报告。研究团队将以摘要化、通俗易懂的形式，向愿意接收结果的受试者反馈总体、非个体化研究结果。任何出版物、报告或反馈材料均不会披露可识别个人身份的信息。

**9.6 质量控制与保障**

（1）建立独立研究指导委员会或等效研究监督机制，以监督科学有效性、受试者安全、方案实施和整体研究进展。

（2）开展定期内部数据检查和质量控制审查，包括核查CRF、知情同意文件、资格证明文件、干预记录、不良事件记录和结局评估完整性。

（3）接受本机构伦理委员会的持续审查、进展审查和检查，并按要求及时提交安全性报告、方案修正和研究进展报告。
